# MKRN3 regulates the epigenetic switch of mammalian puberty via ubiquitination of MBD3

**DOI:** 10.1093/nsr/nwaa023

**Published:** 2020-02-14

**Authors:** Chuanyin Li, Wenli Lu, Liguang Yang, Zhengwei Li, Xiaoyi Zhou, Rong Guo, Junqi Wang, Zhebao Wu, Zhiya Dong, Guang Ning, Yujiang Shi, Yinmin Gu, Peng Chen, Zijian Hao, Tianting Han, Meiqiang Yang, Wei Wang, Xuehui Huang, Yixue Li, Shan Gao, Ronggui Hu

**Affiliations:** 1 Department of Pediatrics, Ruijin Hospital Affiliated to Shanghai Jiao Tong University School of Medicine, Shanghai 200025, China; State Key Laboratory of Molecular Biology, CAS Center for Excellence in Molecular Cell Institute of Biochemistry and Cell Biology, Chinese Academy of Sciences, Shanghai 200031, China; 2 University of Chinese Academy of Sciences, Beijing 100049, China; 3 CAS Key Laboratory of Computational Biology, CAS-MPG Partner Institute for Computational Biology, Shanghai Institute of Nutrition and Health, Shanghai Institutes for Biological Sciences, University of Chinese Academy of Sciences, Chinese Academy of Sciences, Shanghai 200031, China; 4 College of Life Sciences, Shanghai Normal University, Shanghai 200234, China; 5 Center for Pituitary Tumor, Ruijin Hospital Affiliated to Shanghai Jiao Tong University, Shanghai 200025, China; 6 Shanghai Institute of Endocrine and Metabolic Diseases, Shanghai Key Laboratory for Endocrine Tumors and E-Institute for Endocrinology, Ruijin Hospital, Shanghai Jiao Tong University School of Medicine, Shanghai 200025, China; 7 Division of Endocrinology, Diabetes and Hypertension, Department of Medicine, Brigham and Women's Hospital, Boston, MA 02115, USA; 8 Harvard Medical School, Boston, MA 02115, USA; 9 CAS Key Laboratory of Bio-medical Diagnostics, Suzhou Institute of Biomedical Engineering and Technology, Chinese Academy of Sciences, Suzhou 215163, China; 10 Cancer Center, Shanghai Tenth People's Hospital, School of Medicine, Tongji University, Shanghai 200072, China

**Keywords:** ubiquitin, MKRN3, DNA methylation, MBD3, DNA demethylase, central precocious puberty

## Abstract

Central precocious puberty (CPP) refers to a human syndrome of early puberty initiation with characteristic increase in hypothalamic production and release of gonadotropin-releasing hormone (GnRH). Previously, loss-of-function mutations in human *MKRN3*, encoding a putative E3 ubiquitin ligase, were found to contribute to about 30% of cases of familial CPP. MKRN3 was thereby suggested to serve as a ‘brake’ of mammalian puberty onset, but the underlying mechanisms remain as yet unknown. Here, we report that genetic ablation of *Mkrn3* did accelerate mouse puberty onset with increased production of hypothalamic GnRH1. MKRN3 interacts with and ubiquitinates MBD3, which epigenetically silences *GNRH1* through disrupting the MBD3 binding to the *GNRH1* promoter and recruitment of DNA demethylase TET2. Our findings have thus delineated a molecular mechanism through which the MKRN3–MBD3 axis controls the epigenetic switch in the onset of mammalian puberty.

## INTRODUCTION

Mammalian puberty is a process through which sexual competence and reproduction are attained under the regulation of the anatomic hypothalamic–pituitary–gonadal (HPG) axis [1]. The HPG axis is active during fetal and neonatal stages, but becomes quiescent during childhood, until ultimately reactivated upon pubertal initiation, which is characterized by an increase in the amplitude and frequency of pulsatile hypothalamic gonadotropin-releasing hormone (GnRH) release. The re-emergence of pulsatile GnRH secretion subsequently leads to increases in the secretion of gonadotropins, luteinizing hormone (LH) and follicle-stimulating hormone (FSH) by the anterior pituitary gland and the consequent activation of gonadal function [2]. The HPG axis can also be prematurely activated to initiate gonadotropin-dependent precocious puberty, resulting in central precocious puberty (CPP) that clinically defines those who develop secondary sexual characteristics before the age of 8 years in girls or 9 years in boys. CPP is currently estimated to affect approximately 1 in 5000–10 000 of the population worldwide, resulting from complex interactions among genetic, nutritional, environmental and socioeconomic factors. The dysregulated timing of puberty is known to associate with risks of many subsequent diseases, e.g. earlier age of menarche in girls is associated with increased risks of breast cancer, endometrial cancer, obesity, type 2 diabetes and cardiovascular disease, indicating the critical importance of the delicate but as-yet-unknown mechanisms that regulate human puberty [3,4]. Meanwhile, epigenetic reprogramming has been proposed to play pivotal roles in the expression of puberty-related genes in the control of puberty initiation and development, with detailed mechanisms as yet incompletely understood [5–7].

A recent study has revealed mutations or deletions in the human *MKRN3* (Makorin ring finger protein 3) gene in familial CPP cohorts, which was estimated to contribute to ∼30% of familial CPP cases worldwide [8]. As many of these mutations might disrupt the MKRN3 structure and impair its E3 ubiquitin ligase activity, MKRN3 was proposed to act as a ‘brake’ or repressor of GnRH production in regulating mammalian puberty. However, it remains totally unclear how the disease-causing mutations or deletions in the *MKRN3* gene lead to premature GnRH production and release [9].

DNA methylation and demethylation are catalysed by DNA methyltransferases (DNMTs) and demethylases (human ten-eleven translocation methylcytosine dioxygenases, TETs), respectively. Such reactions occur at the carbon-5 position of cytosine residues in CpG nucleotides, leading to the formation or decomposition of 5-methylcytosine (5mC), 5-hydroxylmethylcytosine (5hmC) and other derivatives, which constitutes a fundamental epigenetic mechanism that regulates gene expression in mammalian cells [10–13]. Proteins of the methyl-CpG-binding domain (MBD) family were first defined as those that read DNA methylation and recruit chromatin remodelers, e.g. histone deacetylases and methyltransferases, to methylated DNA and repress gene expression [14]. Among MBDs (Methyl-CpG-DNA binding proteins), MBD2 and MBD3 interact with the nucleosome remodeling deacetylase (NuRD/Mi-2) complex members to repress gene expression. However, unlike MBD2 that binds to methylated DNAs and mainly represses gene expression, MBD3 has been shown to bind primarily to promoters, exons and enhancers of actively transcribed genes, suggesting more diverse roles of MBD3 in regulating gene expression [15–18]. MBD3 and some of its isoforms were found to interact with and promote the DNA demethylase activity of human ten-eleven translocation methylcytosine dioxygenase 2 (TET2), which could dynamically and specifically impact the methylation status of gene loci [19]. Mutations within MBD3 were associated with human cancer and neurocognitive disorders, such as autistic spectrum disorders (ASDs) [20,21]. Furthermore, MBD3 has been shown to modulate cell pluripotency, but with opposite effects in different studies [17,22–24]. Such findings, while reminding us of how little we know about the complex mechanisms through which MBD3 exerts its biological roles, may be reconciled by taking into account the possibility that MBD3 could undergo distinct post-translational modifications that might differentially modulate the homeostasis and function of MBD3 in a spatiotemporally controlled manner. However, it remains entirely unknown whether and how such post-translational modifications might occur and regulate MBD3 function in any pathophysiologically meaningful way.

In this work, we generated the first genetic model of human CPP by constructing a *Mkrn3* knockout mouse strain and establishing its role in regulating the onset of puberty and a repressor role in regulating *Gnrh1* expression. We next identified MBD3 as a novel physiological substrate for the E3 ubiquitin ligase activity of MKRN3. MKRN3-mediated ubiquitination was found to disrupt the interactions between MBD3 with 5hmC-containing *GNRH1* promoter or TET2, resulting in the epigenetic silence of *GNRH1* expression. Moreover, it was confirmed in patient-derived cells that the disease-causing mutations indeed impaired the ubiquitination of endogenous MBD3, supporting a strong clinical relevance of our experimental findings.

## RESULTS

### Maternally imprinted Mkrn3 regulates the onset of puberty and represses *Gnrh1* expression in mice

**Figure 1.**
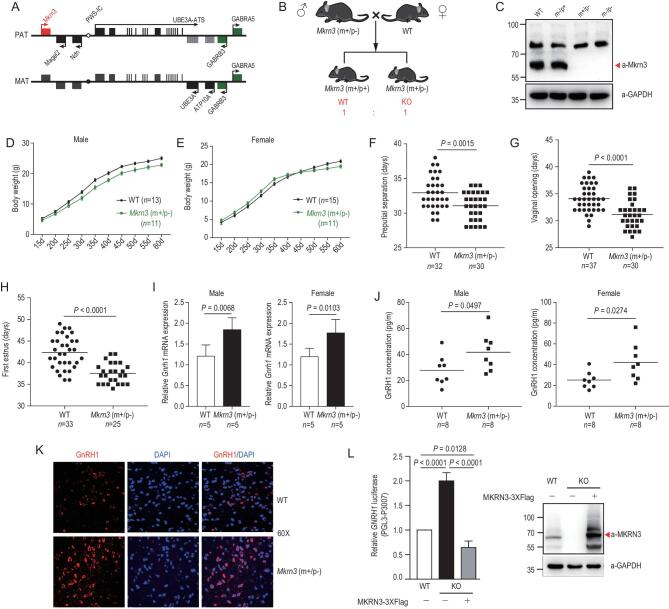
Loss of Mkrn3 in mice phenocopies of human central precocious puberty (CPP), with early puberty onset and increased hypothalamic production of GnRH1. (A) Schematic view of the mouse *Mkrn3* gene locus. *Mkrn3* and the neighboring two genes, *Magel2* and *Ndn*, are expressed only from the paternal allele. PAT, paternal; MAT, maternal. (B) Strategy for the generation of *Mkrn3*-deficient mice. *Mkrn3* is expressed only from the paternally inherited allele. The mating of a heterozygous *Mkrn3 (m+/p−)* male with a wild-type female mouse could generate equal numbers of Mkrn3-deficient and wild-type offspring. (C) Expression of endogenous Mkrn3 protein in hypothalamic extracts from female mice of the indicated *Mkrn3* genotypes at postnatal day 15. p, paternally inherited allele; m, maternally inherited allele. +, wild-type; −, knockout. (D) Body weights of male mice of indicated genotypes from postnatal day 15 to day 60. Wild-type (WT), *n* = 13; Mkrn3-deficient (m+/p−), *n* = 11. (E) Body weight of female mouse from postnatal day 15 to day 60. Wild-type (WT), *n* = 15; Mkrn3-deficient (m+/p−), *n* = 11. (F) Differential preputial separation time of Mkrn3-deficient (m+/p−) and wild-type male mouse. Wild-type (WT), *n* = 32; Mkrn3-deficient (m+/p−), *n* = 30, two-tailed unpaired *t*-test. (G) Differential vaginal opening time of Mkrn3-deficient (m+/p−) and wild-type female mouse. Wild-type (WT), *n* = 37; Mkrn3-deficient (m+/p−), *n* = 30, two-tailed unpaired *t*-test. (H) Differential first estrus time of Mkrn3-deficient (m+/p−) and wild-type female mouse. Wild-type (WT), *n* = 33; Mkrn3-deficient (m+/p−), *n* = 25, two-tailed unpaired *t*-test. (I) Levels of *Gnrh1* mRNA in the hypothalamus of 15-day-old male and female *Mkrn3 (m+/p−)* mice and age-matched wild-type mice, determined by quantitative Polymerase Chain Reaction (qPCR), ribosomal protein L19 was taken as endogenous control. Five mice in each group, two-tailed unpaired *t*-test. (J) Serum GnRH1 concentration of Mkrn3-deficient (m+/p−) and wild-type male/female mice were detected by enzyme-linked immunosorbent assay (ELISA) at postnatal day 15. Wild-type (WT), *n* = 8; Mkrn3-deficient (m+/p−), *n* = 8, two-tailed unpaired *t*-test. (K) The hypothalamus immunofluorescence stain of female Mkrn3 (m+/p−) mice and age-matched wild-type mice using GnRH1 antibody at postnatal day 15 (60×). (L) Luciferase reporter assay to detect the relative activities of the human *GNRH1* promoter in HEK293T cells of indicated genotypes: wild-type, MKRN3 KO or MKRN3 KO with MKRN3 reintroduced. Data are presented as mean ± SD, one-way Analysis of Variance (ANOVA), with Bonferroni post-hoc test, three independent experiments.

The mouse *Mkrn3* gene contains a single exon and resides adjacent to the Prader–Willi Syndrome Imprinting Center (PWS-IC) on chromosome 7, which encodes Mkrn3 protein that shares high sequence homology with other mammalian orthologs (Fig. 1A and Supplementary Fig. 1A). The mouse *Mkrn3* gene was expressed in tissues with a distinct pattern, being most abundant throughout the brain, lower in testis and barely detectible in all other tissues examined (Supplementary Fig. 1B). Mkrn3 protein expression in mouse hypothalamus was found to undergo an age-dependent decrease during development, with a sharp reduction occurring at postnatal day 15 to day 18, coinciding with the time of the first neuroendocrine step of puberty initiation (Supplementary Fig. 1C). Consistently with a previous report [8], the age-dependent decline in *Mkrn3* expression was found to inversely correlate with that of *Gnrh1* in the hypothalamus of mice (Supplementary Fig. 1D).

Mice were genetically modified through introducing a 2-bp deletion in the single exon of the *Mkrn3* gene using the TALEN, transcription activator-like (TAL) effector nucleases, technique (Supplementary Fig. 1E) and a mating of a heterozygous Mkrn3^+/^^–^ male with a wild-type female generated equal numbers of Mkrn3-deficient and wild-type offspring (Fig. 1B). As shown in Fig. 1C, the expression of endogenous Mkrn3 protein was indeed abolished in *Mkrn3*^−/−^ mice or in heterozygotes that harbored wild-type *Mkrn3* only in the maternally inherited allele (*Mkrn3*^m+/p−^). Meanwhile, Mkrn3 was expressed at levels comparable to those in age- and sex-matched littermates that harbored wild-type Mkrn3 either in the paternally inherited allele only (*Mkrn*3^m^^–^^/p+^) or in both alleles (*Mkrn*3^+/+^), suggesting that *Mkrn3* is indeed maternally imprinted in mice. The brain immunostaining showed that Mkrn3 could be detected in the whole hypothalamus of wild- type mice, but little in *Mkrn3*^m+/p−^ mice (Supplementary Fig. 1F).

Phenotypical studies were then performed with the mice. Male mice with *Mkrn3* ablated in the paternally inherited allele (*Mkrn*3^m+/p−^) manifested in reduced body weights when compared to those of the age-matched wild-type ones through postnatal day 15 to day 60 (Fig. 1D). However, the female *Mkrn3*-ablated mice showed higher body weight from postnatal day 15 to day 40, but reduced body weight was observed from postnatal day 45 to day 60 compared to those age-matched wild-type mice (Fig. 1E). The preputial separation time of male *Mkrn3^m+/p^^−^* mice (31.07 ± 0.3649 days) was earlier than that of wild-type mice (32.94 ± 0.4211 days) (Fig. 1F and Supplementary Fig. 1G) and testis hematoxylin-eosin (HE) staining indicated that the stage of elongating spermatids in *Mkrn3^m+/p^^−^* mice comes earlier than that of wild-type mice (Supplementary Fig. 1H). The virginal opening time of female *Mkrn3^m+/p^^−^* mice (31.17 ± 0.4525 days) was earlier than that of wild-type mice (34.11 ± 0.4023 days) (Fig. 1G and Supplementary Fig. 1G). The earlier first time of estrus was observed in *Mkrn3^m+/p^^−^* female mice (37.56 ± 0.4693) compared to that of wild-type mice (42.33 ± 0.6435) (Fig. 1H and Supplementary Fig. 1I). However, the first litter numbers were almost the same regardless of the genotype (Supplementary Fig. 1J). These observations were remarkably reminiscent of the symptoms typically observed in untreated CPP patients.

The mRNA of *Gnrh1* expressed in *Mkrn3*^m+/p−^ mice, measured at the age of 15 days, was approximately 50% higher than that of the wild-type controls (Fig. 1I), while no significant changes were detected in the expression of *Kiss1* or *NKB* (Supplementary Fig. 1K). The levels of GnRH1 in both hypothalamic tissue and serum were significantly higher in *Mkrn3*^m+/p−^ mice than those in control groups (Fig. 1J and Supplementary Fig. 1L). The serum levels of LH and FSH were then measured and both were found to be 1.5- or 2-fold higher in *Mkrn3*^m+/p−^ mice than in wild type (Supplementary Fig. 1M). Immunostaining further indicated that the hypothalamic production of GnRH1 protein was markedly higher in Mkrn3^m+/p−^ mice than in wild type (Fig. 1K). Similar results were also observed with GNRHR (GnRH1 receptor) (Supplementary Fig. 1N). Meanwhile, no obvious changes were observed at the protein level of KISS1 and NKB in Mkrn3^m+/p−^ mice compared to that in wild type (Supplementary Fig. 1O and P).

Luciferase reporter assays further indicated that the transcriptional activation of the human *GNRH1* gene promoter (−3007 to 0 bp) was 100% higher in *MKRN3^−^^/^^−^* HEK293T cells; however, the reintroduction of MKRN3 led to ∼2- to 3-fold reduction in gene expression driven by the *GNRH1* promoter (Fig. 1L and Supplementary Fig. 1Q and R). In GT1–7 cells that were derived from hypothalamic GnRH-positive neurons, mouse Mkrn3 potently repressed *Gnrh1* expression at both the mRNA and the protein levels (Supplementary Fig. 1S). Altogether, these findings established MKRN3 as an authentic repressor of GnRH1 expression at both the cellular and the organism levels.

### CPP-associated mutations compromise the auto-ubiquitination of MKRN3

**Figure 2.**
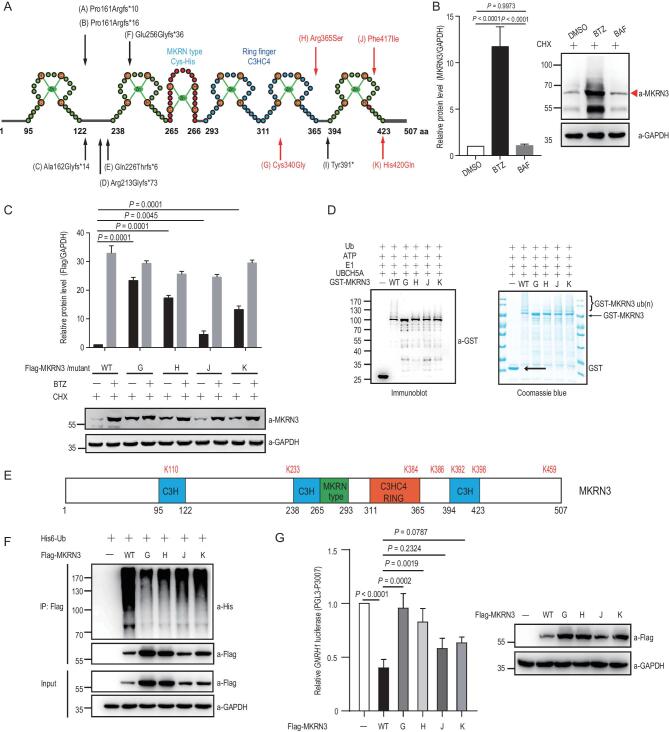
CPP-associated mutations compromise the auto-ubiquitination of MKRN3 and affect its stability. (A) MKRN3 protein structure and mutations previously identified in patients with CPP. Zn, zinc; H, histidine; C, cysteine. Black arrows indicate the location of frameshift mutations; red arrows indicate the location of the missense mutations. (B) Endogenous MKRN3 primarily undergoes proteasome-dependent degradation. BTZ, Bortezomib, a proteasome inhibitor; BAF, Bafilomycin, an autophagy inhibitor; CHX, cycloheximide. Before harvest, HEK293T cells were treated with BTZ (1 μM) or BAF (20 nM) as well as CHX (100 μg/ml) for 6 hrs. Data are presented as mean ± SD, one-way ANOVA, with Bonferroni post-hoc test, three independent experiments. (C) Wild-type MKRN3 protein was less stable than four mutants bearing CPP-associated missense mutations. Flag-tagged MKRN3 proteins, wild-type or with indicated mutations, were expressed in HEK293T *MKRN3*^−^^/^^−^ cells. Cells were treated with CHX and with or without BTZ for 8 hrs before harvest for immunoblotting analysis. Data are presented as mean ± SD, one-way ANOVA, with Bonferroni post-hoc test, three independent experiments. (D) Disease-associated missense mutations of MKRN3 attenuated their auto-ubiquitination in an *in vitro* ubiquitination assay, as detected by immunoblotting with a Gst antibody (left panel) and Coomassie Blue stain (right panel). (E) Schematic distribution of the seven Lys residues in MKRN3 auto-ubiquitination sites, identified with MKRN3 recovered from the auto-ubiquitination assay *in vitro* and then subjected to mass-spectrum analysis. (F) Ubiquitination of wild-type MKRN3 protein was more significant than those of the disease-associated mutants in HEK293T *MKRN*3^−^^/^^−^ cells. Cells were transfected with plasmids encoding His6-Ub and Flag-tagged MKRN3, wild-type or with the indicated missense mutations. MKRN3 proteins were immunoprecipitated using anti-Flag beads followed by immunoblotting with anti-His to detect ubiquitination signals. (G) Mutations of MKRN3 compromised its inhibitory effect on *GNRH1* promoter activity. HEK293T *MKRN*3^−^^/^^−^ cells were co-transfected with the *GNRH1*-luciferase reporter and plasmids encoding wild-type MKRN3 or the indicated mutants. Data are presented as mean ± SD, one-way ANOVA, with Bonferroni post-hoc test, three independent experiments.

MKRN3 is highly conserved in mammals, especially in its RING finger C3HC4 motif, which likely harbors the active site for an E3 ubiquitin ligase (Supplementary Fig. 1A). Currently, multiple mutations of MKRN3, either missense or frameshift, have been found in individuals with CPP and were proposed to compromise its E3 ligase activity, since individuals harboring missense or frameshift mutations manifested similar symptoms (Fig. 2A). Specifically, four of these missense mutations in *MKRN3*, indicated in red, were located within the zinc finger or RING finger motifs that were known to be essential for its E3 ubiquitin ligase activity (Fig. 2A). We next examined whether and how these CPP-associated mutations in *MKRN3* might indeed impact the function of MKRN3. As shown in Fig. 2B, levels of the endogenous MKRN3 protein were increased upon treatment with the proteasomal inhibitor Bortezomib (BTZ) but not the autophagy inhibitor Bafilomycin A1 (BAF) in HEK293T cells, while all *de novo* protein synthesis was inhibited by cycloheximide (CHX). This result clearly suggested that the degradation of endogenous MKRN3 protein was proteasome-dependent, rather than through autophagy. Compared with wild-type MKRN3, all the four CPP-causing mutants showed significantly (3- to 8-fold) higher stability when ectopically expressed in HEK293T *MKRN3^−^^/^^−^* cells or *Mkrn3^m+/p−^* MEF cells (Fig. 2C and Supplementary Fig. 2A and B). These data revealed that auto-ubiquitination is another layer of regulation on the homeostasis of MKRN3 and, presumably, its functionality as well.

An *in vitro* ubiquitination system was then assembled to contain ATP, an E1 Ub-activating enzyme (Uba1), MKRN3 (the E3 ligase) and different E2 Ub conjugating enzymes that could support MKRN3 auto-ubiquitination (Supplementary Fig. 2C). GST pull-down assay indicated that recombinant MKRN3 protein directly interacted with UBCH5A, a member of the UBCH5 subfamily of E2s that could also support MKRN3 auto-ubiquitination (Supplementary Fig. 2D). UBCH5A was thus selected for the *in vitro* ubiquitination assay. As shown in Supplementary Fig. 2E, after 15 min of incubation, diagnostic ladders of bands appeared above unmodified MKRN3 in both immunoblots and Coommassie Blue gel staining, which were representative of ubiquitinated MKRN3, as they could be efficiently eliminated by treatment with USP2cc, the catalytic core of human deubiquitinase USP2. It was thus clear that wild-type MKRN3, like many other RING-type E3 ligases [25], could undergo auto-ubiquitination. When similar *in vitro* ubiquitination reactions were performed with either wild-type MKRN3 or the indicated mutants, it was evident that all four disease-causing mutations compromised the auto-ubiquitination of MKRN3 (Fig. 2D). Further mass-spectrum analysis revealed the side chains of multiple Lys (K) residues as the sites for MKRN3 auto-ubiquitination (Fig. 2E). As shown in Fig. 2F, the CPP-associated mutants did undergo less, although varying, ubiquitination than wild-type MKRN3. These data suggest that the CPP-causing mutations might stabilize MKRN3 protein through impairing their E3 ligase activity, resulting in less auto-ubiquitination and slower proteasome-mediated degradation. Compared to wild-type MKRN3, the introduction of CPP-causing MKRN3 mutants also led to weaker suppression of the transcriptional activity of the *GNRH1* promoter (Fig. 2G). There thus appeared to be an association between the E3 ligase activity of MKRN3 and its ability to suppress *GNRH1*-promoter-driven gene expression.

### MKRN3 interacts with and ubiquitinates MBD3

**Figure 3.**
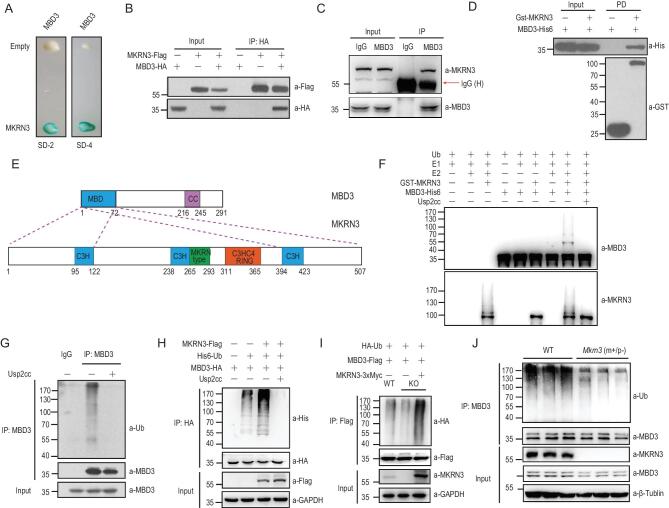
Identification of MBD3 as a novel physiological substrate for the E3 Ub ligase activity MKRN3. (A) Yeast two-hybrid (Y2H) screening identified MBD3 as an interacting partner for MKRN3. MKRN3 was used as a bait, SD-2, deficient in *Leu* and *Trp*; SD-4, deficient in *Ura*, *His*, *Leu* and *Trp*. (B) MKRN3-Flag and MBD3-HA formed a complex in HEK293T cells, as detected by a co-immunoprecipitation assay performed using anti-Flag antibody, followed by immunoblotting with anti-HA and anti-Flag antibodies. (C) Endogenous MKRN3 and MBD3 form a complex in HEK293T cells, as demonstrated by a co-immunoprecipitation assay using anti-IgG or anti-MBD3, followed by immunoblotting with anti-MKRN3 or anti-MBD3 antibodies. (D) GST pull-down assays indicated that MKRN3 protein interacted directly with His6-tagged MBD3. PD, GST pull-down. (E) The MBD domain of MBD3 interacted with both N- and C-termini of MKRN3 (see more details in Supplementary Fig. 3A and B). (F) MKRN3 ubiquitinates MBD3 *in vitro*. An *in vitro* ubiquitination assay was carried out using the recombinant proteins MBD3, UBA1 (E1), UBCH5A (E2) and MKRN3, together with the indicated components. Both MBD3 ubiquitination and MKRN3 auto-ubiquitination were detected. Usp2cc, the catalytic core of human deubiquitinase Usp2. (G) Endogenous MBD3 was ubiquitinated in HEK293T cells. MBD3 was immunoprecipitated from HEK293T cells using anti-MBD3 and immunoblotted with anti-Ub. (H) MBD3-HA was ubiquitinated by Flag-MKRN3 in HEK293T cells. Cells were co-transfected with the indicated combinations of plasmids; 24 hrs later, cell lysates were immunoprecipitated with anti-HA beads and subjected to immunoblotting analysis. (I) MBD3 ubiquitination is reduced in HEK293T *MKRN*3^−^^/^^−^ cells compared to wild-type HEK293T cells. Reintroduced MKRN3 can rescue MBD3 ubiquitination. *MKRN*3^−^^/^^−^ or wild-type HEK293T cells were co-transfected with HA-Ub, MBD3-Flag and/or MKRN3-3xMyc as indicated. (J) MBD3 ubiquitination is reduced in *Mkrn3(m+/p−)* mouse brains at postnatal day 15 compared to age-matched wild-type littermates. Endogenous MBD3 proteins of whole-brain lysates from wild-type or *Mkrn3 (m+/p−)* mice were immunoprecipitated using anti-MBD3, followed by immunoblotting with anti-Ub. Three mice in each group.

Yeast two-hybrid (Y2H) screening was performed to identify a potential substrate(s) for the E3 ligase MKRN3. MKRN3 was used as the bait to screen potential MKRN3-interacting proteins from a Y2H prey library containing over 15 000 human open reading frames (ORFs) [26]. Positive colonies were selected for sequencing and MBD3 emerged as a potential MKRN3-interacting partner. An MKRN3–MBD3 interaction was confirmed by survival assay in yeast SD-Leu-Trp-His-Ura (SD-4) selection medium, as well as a plate-based staining assay of β-galactosidase activities (Fig. 3A). MKRN3 and MBD3 proteins, either endogenously or exogenously expressed, co-immunoprecipitated (Co-IP) with each other, indicating that they could form a complex *in vivo* (Fig. 3B and C). GST pull-down assays showed that MKRN3 directly interacted with MBD3 *in vitro* (Fig. 3D) and both the N- and C-termini of MKRN3 were implicated in the interaction with the MBD domain in MBD3 (Fig. 3E and Supplementary Fig. 3A and B). MKRN3 and MBD3, tagged with green fluorescence protein and red fluorescence protein, respectively, co-localized in the nuclear region of the cell (Supplementary Fig. 3C). Interestingly, the four CPP-causing mutations in MKRN3 seemed to have little or no effect on the interaction between MKRN3–MBD3 (Supplementary Fig. 3D), suggesting that these mutations might cause CPP through mechanisms other than directly affecting the MKRN3–MBD3 interaction.

*In vitro* ubiquitination reactions were assembled containing recombinant MKRN3 and MBD3 proteins as E3 ligase and substrate, respectively. As shown in Fig. 3F, MKRN3 was able to conjugate poly-Ub chains onto MBD3 *in vitro*. Endogenous MBD3 in HEK293T cells also underwent ubiquitination, as assessed by anti-MBD3-based immunoprecipitation and subsequent immunoblotting analysis using anti-Ub (Fig. 3G). An *in vivo* ubiquitination assay was then performed with tagged MKRN3 and MBD3 in HEK293T cells (Fig. 3H). While genetic ablation of MKRN3 markedly reduced the ubiquitination of endogenous MBD3, the reintroduction of MKRN3 into *MKRN3*^−/−^ HEK293T cells led to much increased ubiquitination of MBD3 (Fig. 3I). Consistently, endogenous MBD3 underwent less ubiquitination in the brains of *MKRN3*^m+/p−^ mice at the age of 15 days compared to that of age-matched wild-type littermates (Fig. 3J).

Altogether, these data suggested that MBD3 emerged as a physiological substrate for MKRN3.

### MKRN3 mediates the non-proteolytic ubiquitination of MBD3 at multiple sites

**Figure 4.**
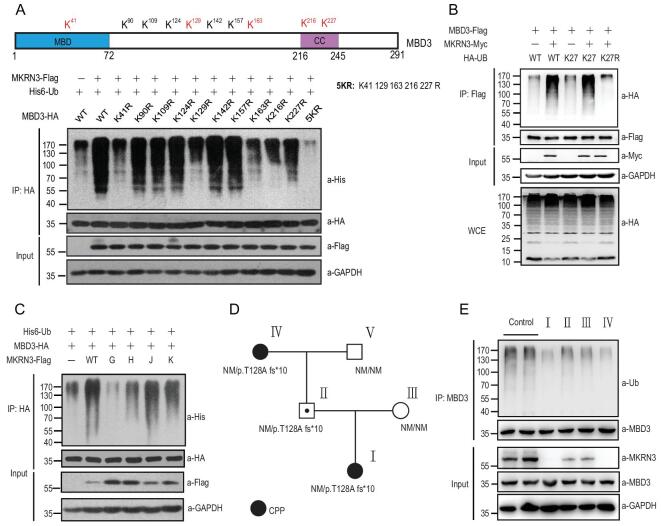
MKRN3 ubiquitinates MBD3 on multiple Lys residues with Lys27 ubiquitin linkages and mutation of MKRN3 reduces MBD3 ubiquitination. (A) Schematic distribution of the 10 sites (Lys residues) of MKRN3-mediated ubiquitination of human MBD3. MBD3 proteins were recovered from the *in vitro* ubiquitination assay and subjected to mass-spectrum analysis to map the ubiquitination sites. Five Lys residues (41, 129, 163, 216 and 227, shown in red) turned out to be the major sites for MKRN3-mediated ubiquitination of MBD3 in HEK293T cells. 5KR, the mutant simultaneously bearing K-to-R mutations in all the five Lys residues in MBD3. (B) MKRN3 ubiquitinates MBD3 with non-proteolytic Lys27 ubiquitin linkages. HEK293T *MKRN*3^−^^/^^−^ cells were co-transfected with MKRN3-Myc, MBD3-Flag and HA-UB (WT, mutated at all Lys residues except Lys27 (K27) or mutated only at Lys 27 (K27R)). Cell lysates were immunoprecipitated with anti-Flag beads followed by immunoblotting using anti-HA to detect MBD3 ubiquitination. (C) Disease-associated mutations in MKRN3 compromised its E3 ligase activity towards MBD3 in HEK293T *MKRN*3^−^^/^^−^ cells. Cells were transfected with His6-Ub, MBD3-HA and wild-type MKRN3 or the indicated mutants, followed by IP using anti-HA beads and anti-His to detect MBD3 ubiquitination. (D) Pedigree of a Chinese family with a novel p.T128Afs*10 mutation in *MKRN3*. Squares indicate male family members; circles, female family members; black, individuals with CPP; symbol with black dot inside, asymptomatic carriers; NM, no mutation allele. (E) Reduced MBD3 ubiquitination in immortalized lymphoblasts from individuals with CPP compared to unaffected family members and healthy controls. Blood samples were collected from the indicated family members and two healthy age-matched girls, followed by EBV infection to immortalize the cells. Ubiquitination states of endogenous MBD3 proteins were assayed by immunoprecipitation using anti-MBD3 followed by immunoblotting with anti-Ub.

Mass-spectrum analysis revealed that MKRN3 conjugated poly-Ub chains onto the side chains of 10 Lys residues in MBD3 (K41, K90, K109, K124, K129, K142, K157, K163, K216 and K227) *in vitro*. Further *in vivo* ubiquitination assays indicated that five Lys residues (K41, K129, K163, K216 and K227) were major sites in MBD3 for MKRN3-mediated ubiquitination, as these MBD3 mutants bear Lys-to-Arg (K-to-R) substitutions at the indicated positions, individually or simultaneously on all five sites that generated MBD3_5KR_, were less ubiquitinated in HEK293T cells expressing Flag-tagged MKRN3 (Fig. 4A). These five ubiquitination sites were conserved in the MKRN3 proteins of different origins (Supplementary Fig. 4A).

Interestingly, MKRN3-mediated ubiquitination of MBD3 seemed to have little or no effect on the stability of MBD3 (Fig. 4A). An *in vivo* ubiquitination assay with HA-tagged Ub that was either wild type, a Ub mutant that bore K-to-R mutations at all seven Lys (K) residues (KO) or those bearing K-to-R mutations at all Lys residues except one intact Lys only at the indicated residue (K6, K11, K27, K29, K33, K48, K63) indicated that MKRN3-conjugated poly-Ub chains on MBD3 were mainly in K27 linkage (Supplementary Fig. 4B), one type of poly-Ub chain that typically does not lead to degradation [27–30]. This was further confirmed by an *in vivo* ubiquitination assay with an HA-tagged Ub mutant bearing a K-to-R mutation at Lys27 (K27R), which was only poorly conjugated by MKRN3 onto MBD3 (Fig. 4B).

### CPP-causing mutations in MKRN3 impair its ligase activity towards MBD3

Having discovered that CPP-causing mutations compromised the auto-ubiquitination of MKRN3 (Fig. 2F), we went on to ask whether the ubiquitination of MBD3 might also be affected. As shown in Fig. 4C and Supplementary Fig. 4C, all four CPP-causing mutants conjugated poly-Ub chains onto MBD3 much less efficiently than wild-type MKRN3 both *in vitro* and *in vivo*. Similar results were also observed with seven disease-causing truncated mutations of MKRN3 (Fig. 2A and Supplementary Fig. 4D). These data strongly suggested that the disease-causing mutations could indeed impair the E3 ligase activity of MKRN3 and had little or no effect on the stability of MBD3 protein.

### MBD3 ubiquitination is also compromised in cells derived from CPP patients

Recently, a novel mutation (exon1: c.379_398del: p.T128Afs*10) was found in the *MKRN3* gene of a proband with CPP and the immediate family members (Fig. 4D and Supplementary Fig. 4E; see more details in Supplementary Table 1). The proband was a girl (I) with a heterozygous *MKRN3* mutation; her father (II) was a carrier of the mutant allele with no history of CPP; the mother (III) was unaffected; the paternal grandmother (IV) had a history of CPP, whereas the paternal grandfather was unaffected (V). Blood samples were collected from the members of this family, with genomic DNA extracted and subjected to exon sequencing of MKRN3. Additionally, blood samples were also collected from two healthy children with wild-type MKRN3 from unrelated families. The B lymphocytes of the collected samples were then separated and immortalized using an Epstein–Barr virus (EBV)-based approach. An *in vivo* ubiquitination assay was then performed to determine the ubiquitination states of endogenous MBD3 in the cells derived from the patients with CPP, unaffected family members or unrelated apparently healthy controls. As shown in Fig. 4E, MBD3 in cells derived from the CPP patients (I, IV) underwent less ubiquitination than those from the unaffected family members (II, III) or healthy controls.

### MKRN3-mediated MBD3 ubiquitination suppresses *GNRH1* expression

**Figure 5.**
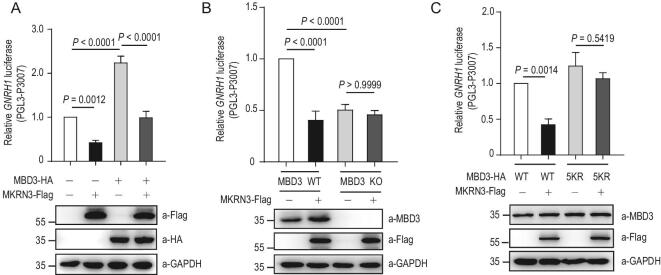
MKRN3-mediated ubiquitination represses MBD3-activated *GNRH1* transcription. (A) MKRN3 inhibits MBD3-activated *GNRH1* promoter activity in luciferase assays. HEK293T cells were co-transfected with the indicated vectors and luciferase activity was measured 48 hrs later. Data are presented as mean ± SD, one-way ANOVA, with Bonferroni post-hoc test, three independent experiments. (B) Endogenous MBD3 was required for MKRN3-mediated repression on *GNRH1* transcription. HEK293T cells (wild-type or *MBD3*^−^^/^^−^) were transfected with vectors as indicated and luciferase activities were measured 48 hrs later. Data are presented as mean ± SD, one-way ANOVA, with Bonferroni post-hoc test, three independent experiments. (C) MKRN3-mediated ubiquitination impaired MBD3-activated transcription driven by the *GNRH1* promoter. MKRN3 and MBD3 double knockout (MKRN3^-/-^ MBD3^-/-^) HEK293T cells were co-transfected with the *GNRH1* luciferase reporter plasmids and plasmids expressing MKRN3 and wild-type or 5KR (five Lys-to-Arg) MBD3; luciferase activity was measured 48 hrs later after transfection. Data are presented as mean ± SD, one-way ANOVA, with Bonferroni post-hoc test, three independent experiments.

To further address the functional consequences of this newly discovered MKRN3–MBD3 interaction, we next assessed the effects of MBD3 and MKRN3 on *GNRH1* expression. As shown in Fig. 5A, a luciferase reporter assay indicated that exogenous expression of MBD3 increased *GNRH1* promoter-driven transcription by 2.2-fold over control, suggesting an activating effect of MBD3 on *GNRH1* transcription. However, such a transcription-activating effect by MBD3 was markedly reduced when MKRN3 was co-introduced into the cells, suggesting that MKRN3 could suppress MBD3-activated *GNRH1* transcription. We next asked whether endogenous MBD3 was required for the inhibitory role of MKRN3 on *GNRH1* promoter activity, by constructing MBD3-deficient (*MBD3*^−/−^) HEK293T cells (Supplementary Fig. 5A). As shown in Fig. 5B, MKRN3 repressed the *GNRH1* promoter-driven transcription of firefly luciferase only in HEK293T cells that expressed endogenous MBD3 but not in those cells (*MBD3*^−/−^) deficient of endogenous MBD3, strongly suggesting a requirement for endogenous MBD3 for MKRN3 to repress *GNRH1* transcription.

Interestingly, MBD3_5KR_, the MBD3 mutant that bore Lys-to-Arg (K-to-R) substitutions at all five major sites for MKRN3-mediated ubiquitination, was able to activate *Gnrh1* promoter-driven transcription as efficiently as wild-type MBD3. However, unlike wild-type MBD3, transcriptional activation by MBD3_5KR_ was no longer susceptible to MKRN3-mediated repression (Fig. 5C), suggesting that MKRN3-conjugated poly-Ub chains at the five sites in MBD3 were primarily responsible for mediating the suppressing effect of MKRN3 on *GNRH1* transcription. As shown in Fig. 2G, 4C and Supplementary Fig. 4C, the four CPP-causing MKRN3 missense mutants, which poorly ubiquitinated MBD3, only exhibited a marginal inhibitory effect on MBD3-activated *GNRH1* expression when compared to that by wild-type MKRN3, suggesting a potential link between impaired MBD3 ubiquitination and weakened suppression of GnRH1 production in CPP patients.

### MKRN3-mediated MBD3 ubiquitination promotes DNA methylation through disrupting the binding of MBD3 to 5hmC-containing DNA or the recruitment of TET2 to the *GNRH1* promoter

**Figure 6.**
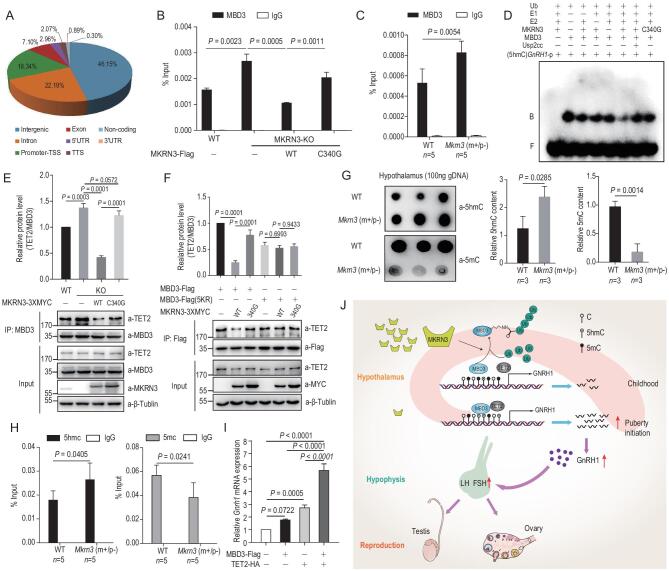
MKRN3–MBD3–TET2 axis regulates the methylation status of mammalian genome, including *GNRH1* promoter. (A) ChIP-seq analysis identified MBD3-binding regions in the human genome. Wild-type HEK293T cells were subjected to ChIP-seq analysis, using anti-MBD3 to enrich DNA-fragment complexes with endogenous MBD3. Percentages shown in the chart indicate the relative abundance of MBD3-bound regions in different structural parts of the genome. (B) MKRN3 inhibits the binding of endogenous MBD3 to the *GNRH1* promoter in a manner depending on its E3 ligase activity. ChIP-qPCR assays were performed with HEK293T cells, wild-type or *MKRN3*^−^^/^^−^ that were transfected with or without wild-type or C340G mutant MKRN3. Data are presented as mean ± SD, one-way ANOVA, with Bonferroni post-hoc test, three independent samples in each group. (C) Loss of Mkrn3 promotes the binding of endogenous MBD3 to the *Gnrh1* promoter in primarily dissected neurons from mouse hypothalamus at postnatal day 15. ChIP-qPCR assays were performed in primarily dissected neurons from the hypothalamus of wild-type and *Mkrn3 (m+/p−)* mice. Data are presented as mean ± SD, two-tailed unpaired *t*-test, five samples in each group. (D) MKRN3-mediated ubiquitination compromised the affinity of recombinant MBD3 to 5hmC (5-hydroxymethylcytosine)-containing DNA sequences derived from the human *GNRH1* promoter. EMSA was performed using −1716 to −1541 bp of the *GNRH1* promoter with all cytosines substituted by 5hmC as a probe, labeled with γ-^32^P. Recombinant MBD3 was subjected to *in vitro* ubiquitination under varying conditions, then immunoprecipitated with anti-Flag beads for purification before using in the EMSA assay. B, bound probe; F, free probe. (E) MKRN3 impairs MBD3–TET2 interaction in HEK293T cells. Wild-type or *MKRN*3^−^^/^^−^ cells were transfected with Myc-tagged wild-type or C340G mutant MKRN3; 48 hrs later, cell lysates were immunoprecipitated with anti-MBD3 antibody, and TET2 and other proteins as indicated were detected by immunoblotting. Data are presented as mean ± SD, one-way ANOVA, with Bonferroni post-hoc test, three independent experiments. (F) MKRN3-mediated ubiquitination disrupts MBD3–TET2 interaction in HEK293T cells. MKRN3/MBD3 double knockout (*MKRN*3^−^^/^^−^*MBD3*^−^^/^^−^) HEK293T cells were co-transfected with either wild-type or C340 mutant MKRN3-3xMyc and Flag-tagged wild-type or 5KR mutant MBD3; 48 hrs later, cell lysates were immunoprecipitated with anti-Flag beads and subjected to immunoblotting with indicated antibodies. Data are presented as mean ± SD, one-way ANOVA, with Bonferroni post-hoc test, three independent experiments. (G) Genetic ablation of Mkrn3 reduced 5mC content and increased 5hmC content in mouse hypothalamic genomic DNA. A dot-blotting assay was performed with genomic DNA extracted from the hypothalamus of wild-type or *Mkrn3 (m+/p−)* mice at postnatal day 15, using anti-5mC or anti-5hmC antibodies. For each sample, 100 ng genomic DNA was loaded onto the nitrocellulose membrane. Three mice in each group, two-tailed unpaired *t*-test. (H) Mkrn3 deficiency was associated with increased 5hmC and decreased 5mC in the *Gnrh1* promoter of the mouse hypothalamus. Genomic DNA was isolated from hypothalamus of wild-type and *Mkrn3 (m+/p−)* mice at postnatal day 15 and immunoprecipitated with anti-5hmC or 5mC antibodies, followed by qPCR using primers for the *Gnrh1* promoter. Three mice in each group, two-tailed unpaired *t*-test. (I) MBD3 and TET2 synergistically activate *Gnrh1* transcription. GT1-7 cells were transfected with MBD3, TET2 or both followed by qPCR analysis of *Gnrh1* expression. GAPDH was taken as the endogenous control, data are presented as mean ± SD, one-way ANOVA, with Bonferroni post-hoc test, three independent experiments. (J) A model depicting how the MKRN3–MBD3–TET2 axis might regulate the timing of mammalian puberty.

To further pinpoint how MKRN3-mediated MBD3 ubiquitination might regulate gene expression, a ChIP-seq assay was performed with wild-type HEK293T cells, using anti-MBD3 antibody. As shown in Fig. 6A, by classifying the reads from MBD3-bound DNA, MBD3-bound DNAs were all over different structural regions in the human genome, with 46.15% in intergenic regions, 22.19% in introns, 18.34% in promoters, 7.10% in exons, 2.96% in 5′-UTRs and 2.07% in transcription termination sites. These data were consistent with the previous reports that MBD3, unlike MBD2, was not enriched in non-coding chromosomal regions [15–18,31,32]. The MBD3-bound genomic loci significantly overlapped with 5hmC-rich or TET2-bound (82.5%) regions, as determined by the cross-analyses of previously published ChIP-seq data for MBD3 and TET2 (see additional details in the ‘Methods’ section), suggesting possible associations between MBD3 and TET2 in activating the transcription of a large set of genes in mammalian genomes (Supplementary Fig. 6A). Pathway analyses further indicated that many development- or reproduction-related pathways were enriched in these MBD3-bound hits (Table 1). Notably, a fragment of sequence corresponding to the human *GNRH1* promoter was identified in these MBD3-bound peaks (Supplementary Fig. 6B). Subsequently, ChIP-qPCR (quantitative PCR) assays were carried out with HEK293T or *MKRN3^−^^/^^–^* HEK293T-expressing wild-type MKRN3 or the C340G mutant, to determine whether endogenous MBD3 protein indeed bound to the human *GNRH1* promoter region. Using primers (F2R2) to amplify DNA spanning −1716 to −1541 bp of the *GNRH1* promoter, in *MKRN3^−^^/^^−^* HEK293T cells, anti-MBD3 antibodies enriched DNAs from the *GNRH1* promoter to an ∼1.7-fold greater extent than from wild-type HEK293T cells; however, such an effect was completely reversed when wild-type MKRN3 was reintroduced into *MKRN3^−^^/^^−^* cells, but not the enzymatically dead MKRN3 (C340G) mutant (mutant H) (Fig. 6B and Supplementary Fig. 6C). In mouse, MBD3 seemed to preferentially bind to the −1000 to −1 region of the *Gnrh1* promoter (using primers f1r1 for −507 to −388 bp) in GT1–7 cells (Supplementary Fig. 6D). In primarily dissected hypothalamic neurons of wild-type C57BL/6 mice, the binding of MBD3 to the *Gnrh1* promoter was substantially increased during development, while Mkrn3 expression declined rapidly (Supplementary Fig. 6E, 1C and D). Loss of Mkrn3 promotes the binding of endogenous MBD3 to the *Gnrh1* promoter at postnatal day 15 (Fig. 6C). These data strongly suggested that an intact E3 ligase activity of MKRN3 towards MBD3 negatively regulates MBD3 binding to the *GNRH1* promoter. Electrophoretic mobility shift assays (EMSA) were also performed with recombinant human MBD3 protein and DNA probes that had sequences derived from the human *GNRH1* promoter (−1716 to −1541 bp) with unmodified (C), 5′-methylated (5mC) or 5′-hydroxymethylated Cytosine (5hmC). As shown in Supplementary Fig. 6F, MBD3 preferentially bound to 5hmC-containing DNA, with less binding to unmodified DNA and no binding to 5mC-containing DNA. The specificity of MBD3 binding to the *GNRH1* promoter was further confirmed, as the binding of MBD3 to the ^32^P-labeled probe could be effectively competed with by unlabeled 5hmC-containing DNA of the same sequence and, to a lesser extent, by the same amount of unmodified DNA, but almost not at all by 5mC-containing DNA in 10-fold excess (Supplementary Fig. 6F). These results strongly supported the notion that endogenous MBD3 might predominantly bind to 5hmC-containing DNA. As shown in Fig. 6D, the binding of MBD3 to the 5hmC-containing DNA probe of the sequence derived from the *GNRH1* promoter was markedly compromised upon MKRN3-mediated ubiquitination *in vitro.*

**Table 1.** Pathway analyses of development- or reproduction-related MBD3-bound genes.

Pathway	Gene_enriched	Count
**Sex_reproduction_pathways**		
GnRH-signaling pathway	*GNRH1,KRAS,PRKCB*	3
Estrogen-signaling pathway	*KRAS,PIK3CB*	2
Ovarian steroidogenesis	*CYP1B1,INSR*	2
Progesterone-mediated oocyte maturation	*KRAS,CDC27,PIK3CB*	3
Prolactin-signaling pathway	*CCND1,KRAS,SOCS5,PIK3CB*	4
Oxytocin-signaling pathway	*CCND1,KRAS,PRKCB,CDKN1A,PRKAB2,PIK3CB,KCNJ4*	7
**Skeleton_related_pathways**		
Osteoclast differentiation	*TAB2,FYN,PIK3CB*	3

Previously, it was reported that MBD3 interacted with TET2 and promoted the demethylase activity of TET2 [19]. Consistently, TET2 seemed to strongly interact with MBD3 in HEK293T cells with much stronger affinity, compared to TET1 or TET3 (Supplementary Fig. 6G). Additional immunoprecipitation assay revealed the C-terminal region of TET2 (residues 1099–1912) and the C-terminal region of MBD3 (residues 91–291) as the interacting domains (Supplementary Fig. 6H and I). As shown in Fig. 6E, Co-IP experiments with anti-MBD3 indicated that endogenous MBD3 protein indeed interacted with TET2 in both WT and *MKRN3*^−/−^ HEK293T cells, but the MBD3-TET2 interaction appeared to be significantly stronger in the absence of endogenous MKRN3. Furthermore, this interaction was much reduced by the reintroduction of wild-type MKRN3, but not by the enzymatically dead MKRN3 (C340G, mutant H). Remarkably, in *MKRN3*^−/−^*MBD3*^−/−^ cells expressing MBD3_5KR_ (which was a defect for MKRN3-mediated ubiquitination), the MBD3–TET2 interaction was not affected, regardless of the presence of either wild-type MKRN3 or its C340G mutant (Fig. 6F).

Taken together, MKRN3-mediated MBD3 ubiquitination seemed to negatively regulate the affinity of MBD3 for both 5hmC-containing DNA and TET2 demethylase. Obviously, when endogenous MKRN3 is physiologically silenced after puberty

initiation, or its functionality is pathologically compromised by CPP-causing mutations, the MBD3 interaction with TET2 and 5hmC DNA could promote the demethylation of cytosine in the *GNRH1* promoter, activating *GNRH1* transcription.

### MKRN3 deficiency promotes DNA demethylation in the hypothalamus of mice

The contents of 5hmC within genomic DNAs (gDNA) in mouse hypothalamus increased significantly from postnatal day 9 to day 30, while *Mkrn3* expression decreased (Supplementary Fig. 6J and 1D). Compared to that of the wild-type mice, genetic ablation of *Mkrn3* seemed to markedly (by 2- to 3-fold) increase the hypothalamic 5hmC contents from *Mkrn3*^m+/p−^ mice while reducing rhw 5mC content (by 4- to 5-fold) in genomic DNA, as revealed by DNA dot-blotting assay using anti-5mC or anti-5hmC antibodies (Fig. 6G). To more specifically determine the effect of Mkrn3 on the methylation status of the *Gnrh1* promoter, genomic DNA was extracted from the hypothalamus of 15-day-old wild-type and *Mkrn3*^m+/p−^ mice and subjected to mDIP (methylated DNA immunoprecipitation) using antibodies that specifically recognize and enrich either 5mC or 5hmC-containing DNAs. Quantitative PCR (qPCR) was then performed with the enriched DNA, using primers to amplify −822 to −659 bp of the *Gnrh1* promoter, to assess the effect of hypothalamic Mkrn3 deficiency on the relative 5mC and 5hmC content in mouse hypothalamus. As shown in Fig. 6H, Mkrn3 deficiency resulted in an ∼50% increase in the 5hmC content and a 35% decrease in the 5mC content of the *Gnrh1* promoter in the mouse hypothalamus. Consistently, *MKRN3*^−/−^ HEK293T cells also showed higher 5hmC and lower 5mC content in the *GNRH1* promoter (−1785 to −1541 bp) compared to those of wild-type HEK293T cells; however, such an effect could be efficiently reversed through the reintroduction of wild-type MKRN3, but not the enzymatically dead MKRN3 (C340G, mutant H) (Supplementary Fig. 6K). Additionally, MBD3 and TET2 synergistically activated *Gnrh1* expression in the mouse GnRH neuron-derived GT1–7 cells (Fig. 6I). Therefore, MKRN3-mediated ubiquitination did seem to

negatively impact the GnRH production by promoting DNA methylation in mammalian *GNRH1* promoters.

## DISCUSSION

MKRN3 has been proposed to act as an upstream inhibitor of mammalian puberty, but the underlying mechanisms remained undetermined [8,9,33–37]. Recently, hypothalamic microRNA, miR-30b, was found to repress MKRN3 expression through interaction with 3′-UTR of MKRN3 mRNA and blocking miR-30b in juvenile female mice increased MKRN3 gene expression and delayed puberty development, which again underscored the importance of MKRN3 in regulating puberty development [38]. Here, through both the phenotypical characterization of a newly constructed *Mkrn3* knockout mouse strain (Fig. 1D–K and Supplementary Fig. 1G–M) and a direct cell-based assay of the suppressive effect of MKRN3 on *GNRH1* promoter activity (Fig. 1L), our work has first established Mkrn3 as an authentic physiological regulator of puberty onset and suppressor of GnRH1 production in mice. Bessa *et al*. reported that paternally inherited MKRN3 mutations in humans did not totally abolish its puberty brake and the effects can be quite subtle, especially in boys [39], but there seemed to be no obviously sex-dependent differences in the puberty phenotype observed in our mouse study. What might account for this apparent discrepancy between mouse and human studies? First, Mkrn3 was genetically ablated in the mice used in our work, while male patients in above mentioned study were bearing different mutations in the MKRN3 gene; moreover, mice used here were of a relatively pure genetic background (C57), while the human subjects in the above study almost inevitably had heterogeneous genetic backgrounds.

Furthermore, we demonstrated that MKRN3 protein did exhibit E3 ligase activity and undergo auto-ubiquitination followed by proteasome-dependent degradation, both of which could be impaired by disease-causing mutations (Fig. 2B–F). These findings revealed auto-ubiquitination as a novel feedback mechanism in controlling the homeostasis of MKRN3 as an important brake in controlling the hypothalamic switch of the puberty regulator.

We next identified methyl-CpG-binding MBD3 protein as a physiological substrate for the E3 ligase activity of MKRN3. We further showed that MKRN3 conjugated non-proteolytic K27-linked poly-Ub chains onto multiple sites in MBD3. Among the ubiquitination sites, K41 resided in the methyl-CpG-binding domain of MBD3 and the ubiquitination of this site might molecularly underscore how MKRN3-mediated ubiquitination disrupts the binding of MBD3 to the 5hmC-containing *GNRH1* promoter. The other major sites for such ubiquitination (K129, K163, K216 and K227) are located primarily in the C-terminal fragment of MBD3—a region directly involved in MBD3–TET2 interaction (Supplementary Fig. 6H and I), suggesting that such polyubiquitination of MBD3 would compromise MBD3–TET2 interaction (Fig. 6E and F). In light of a previous report that MBD3 promotes the DNA oxidase activity of TET2 [19] and our findings that MBD3 and TET2 synergistically activate *GNRH1* gene expression (Fig. 6I), the reduced affinity of MBD3 for TET2 upon MKRN3-mediated ubiquitination would not only result in substantially lower 5hmC content in mammalian genomes, but also compromise the binding of MBD3 to its target loci that include the *GNRH1* promoter. These two mechanisms would concertedly ensure the suppressive effect of MKRN3 on *GNRH1* expression and thereby inhibit pubertal initiation. This MKRN3-imposed repression of *GNRH1* expression would be lost when the action of MKRN3 was impaired through loss-of-function mutations reported in patients with CPP [8,36] (Fig. 2G). Moreover, wild-type MKRN3 failed to suppress *GNRH1* transcription in cells that have endogenous MBD3 genetically ablated (Fig. 5B), suggesting the existence and synergy of the MKRN3–MBD3 complex in regulating the hypothalamic trigger of pubertal initiation. Finally, it was important to note that the ubiquitination-deficient MBD3 mutant (MBD3_5KR_) could activate the *GNRH1* promoter even in the presence of co-expressed MKRN3 (Fig. 5C), directly underscoring the critical importance of MKRN3-mediated ubiquitination of MBD3 in suppressing *GNRH1* expression. Therefore, to precisely program puberty initiation in mammals, the homeostasis and functionality of both MKRN3 and MBD3 need to be delicately controlled in mammalian GnRH neurons to ensure the timely activation of *GNRH1* expression in the hypothalamus (Fig. 6J).

Notably, MBD3 preferentially bound to the *GNRH1* promoter at 5hmC status over that which bears 5mC or is unmodified (Fig. 6D and Supplementary Fig. 6F) and promoted *GNRH1* transcription, suggesting a specific role for MBD3 in promoting rather than suppressing gene expression. This went a step further from the previous findings that MBD3-bound gene loci highly overlapped with 5hmC-rich chromosomal regions [15,17,19,22,40,41], which might mechanistically underlie why MBD3 mutations or aberrances in the 5hmC distribution pattern were associated with human disease, including cancer [21,42–46] and ASD [20,47]. Finally, our work has clearly demonstrated that post-translational modifications such as ubiquitination may distinctly impact the functionality of MBD3 and account for the apparently opposite roles of MBD3 in regulating cell pluripotency [23,24], suggesting yet another layer of regulation for epigenetic programming in mammals with meaningful functional consequences.

## MATERIALS AND METHODS

For details, see supplementary data.

## Supplementary Material

nwaa023_Supplemental_Files
