# Supplementary material for: MKRN3 regulates the epigenetic switch of mammalian puberty via ubiquitination of MBD3
Source: Natl Sci Rev. 2020 Feb 14;7(3):671–85. doi: 10.1093/nsr/nwaa023 (PMC8288866; doi:10.1093/nsr/nwaa023)
Supplement: nwaa023_Supplemental_Files [file nwaa023_supplemental_files.zip › Supplementary_Information_10-01-2020 (2).docx]

**Methods**

**Mice**

C57BL/6 mice were purchased from Shanghai SLAC Laboratory Animal Company and all mice housed in SPF (specific-pathogen-free) facility. *Mkrn3*^+/-^ mice were constructed using TALEN-based approach to create a 2bp deletion (**Fig.S1E**). All animal experiments were performed in compliance with the guidance for the care and use of laboratory animals and were approved by the institutional animal research ethics committee of SIBCB. For genotyping, mouse tail tissues were collected, digested by proteinase K and DNA extracted using phenol–chloroform extraction method. Sequences of the primers used for genotyping were as the following:

Forward primer: 5’- GCCGGGTCTCTTGACGAAGCTGGTCGAGCCATCTC -3’

Reverse primer: 5’- GCCGGCCGGGTCTCGGAGTAGCAGCCGAGCCAATCAGAG -3’

**Mouse phenotype observation**

**Body weight measurement**

Beginning on the postnatal day 15, body weights of the male or female mice with indicated genotypes were recorded every 5 days until postnatal day 60.

**Preputial separation**

Beginning on the postnatal day 23, for male mice the separation of the glans and prepuce were detected by using tweezers at the approximately the same time every day until obviously preputial separation observed, and the date were recorded.

**Vaginal opening**

Beginning on the postnatal day 23, females were examined at approximately the same time each day for the appearance of a small “pin hole” in vagina by tweezers, the vaginal opening date were recorded.

**The first estrus**

For female mice, 3 days after vaginal opening, vaginal smears were collected by wet cotton swab that immersed in PBS at approximately the same time each day, then dried on the slides, methanol fixation for 5 minutes, stained with rapid Wright-Giemsa staining kit (Sangon Biotech, E607315), and detected by BX51 microscope. The estrous cycle were assayed by the cytological appearance as previously described([1](#_ENREF_1)), briefly the estrous cycle of female mice including four stages: 1) Proestrus: cells are almost exclusively clusters of round, well-formed nucleated epithelial cells; 2) Estrus: cells are predominantly cornified squamous epithelial cells, present in densely packed clusters, these cells lack a nucleus and have an angular appearance. 3) Metestrus: small darkly stained leukocytes predominate. Cornified squamous epithelial cells may be observed, often in fragments and the leukocytes are generally neutrophils with sausage-link nuclei which stain very dark purple. 4) Diestrus: the predominant cells are leukocytes and also present will be nucleated epithelial cells and rarely cornified squamous epithelial cells. All the slides with samples from each stage were subjected to microscopy analysis with typical image presented (See Fig.S1I).

**Cell culture and plasmids transfection**

HEK293T and SHY5Y were maintained in Dulbecco’s modified Eagle’s medium (DMEM), GT1-7 cells were maintained in DMEM/F12 (1:1), all supplemented with 10% fetal bovine serum (FBS), 50units/ml penicillin and 50μg/ml streptomycin. DNA Transfections were performed using Lipofectamine 2000 reagent (Invitrogen) as instructed by manufacturer.

**ELISA**

For cells, 48 hours after the GT1–7 cells were transfected; the complete medium was replaced with serum-free DMEM for 24 h to synchronize the cell cycles. 24 hours after co-incubation, the supernatants were harvested and subjected to GnRH1 (Phoenix pharmaceuticals, RK-040-02) concentration analysis according to the manufacturer's recommendations. For serum, the whole blood were collected, precipitation overnight at 4℃ and serum were separation by centrifugation, then assayed for GnRH1, LH (Elabscience, E-EL-M0057c) and FSH (Elabscience, E-EL-M0511c) using ELISA kit according to the manufacturer's recommendations. For hypothalamic tissue, hypothalamus were dissected from mice and lysed in co-IP buffer (50 mM Tris-Cl, pH 7.5, 150 mM NaCl, 5.0 mM EDTA, 1.0% NP-40) supplemented with protease inhibitor cocktail (Roche), sonicated with Bioruptor (UCD-300 ), centrifuged at 13,000 g for 10 min at 4°C, the supernatants were subjected to GnRH1 concentration analysis.

**Plasmids**

All plasmids used in this study were listed in **Table S2**.

**Antibodies and Agarose beads**

Protein G Agarose beads (16-266) were from Merck Millipore. All antibodies used in this study were listed in **Table S3.**

**Quantitative Real-time PCRs (qRT-PCR)**

Total RNAs were extracted from the indicated cells or tissues with Rneasy Plus Kit (Qiagen). CDNAs were synthesized using ReverTra® Ace Qpcr RT Master Mix (Toyobo). Quantitative PCR (Qpcr) Gene amplifications were performed using SYBR Green (Toyobo) on a 7500 real-time PCR machine (ABI), with the relative abundance of each transcript normalized to that of GAPDH/mL19 gene, using the ΔΔCt method([2](#_ENREF_2)). All qPCR data were presented as mean ± SD, n = 3. Sequences for the primers used in this study were listed in **Table S4.**

**Luciferase reporter assays**

To construct the luciferase reporter vector for assaying the transcription driven by human *GNRH1* promoter, DNA fragments of sequence derived from -1 to -3007 region of human *GNRH1* gene was inserted into Pgl3.0-Basic vector to generate P*gnrh1*-luc. HEK293T cells were seeded at 0.5 × 105 cells/well in 24-well plates. After overnight culture, cells were transiently transfected with P*gnrh1*-luc, PRL-TK together with other vectors (MKRN3, MBD3 or their indicated mutants); after 48h transfection, the cells were harvested and lysed with 5X passive buffer and subjected to Dual-Luciferase Reporter assay according to manufacturer’s instruction (Promega).

**Generation of gene-ablated Cell lines**

HEK293T cells were genetically ablated for *MKRN3* and/or *MBD3* genes using CRISPR/Cas9-Grna technique([3](#_ENREF_3)). Briefly, for each gene, several sequence-guiding RNAs (sgRNAs) were designed and first tested for the knockdown efficiencies. Cells transfected with sgRNA-expressing vectors were subjected to selection in puromycin (5μg/ml concentration), with single colonies picked, amplified and subjected to immunoblotting analyses using individual antibodies. Genomic DNAs were extracted and specific target sequences were then amplified followed by Sanger sequencing to confirm the aimed editions in the genomes of each cell line.

**Immunofluorescence microscopy**

Cells or tissue slices were fixed, subjected to incubation with the individual primary antibodies, followed by staining with dye-conjugated secondary antibodies and DAPI, as described before([2](#_ENREF_2)). Images were recorded with microscope BX51 or FV1200, with representative data presented in the main or supplementary figures.

**Expression and purification of recombinant proteins**

GST- or His6 (His)-tag proteins were expressed in the BL21 *E. coli* cells. After IPTG induction, cells were pelleted, lysed and incubated with glutathione or Ni^2+^TA beads to enrich the respective proteins, following procedures described before([2](#_ENREF_2)).

**DNA Dot blot assay**

Genomic DNAs from mouse hypothalamus of indicated ages were extracted with the standard phenol-chloroform approach. Equal amounts of DNAs at the same concentrations were denatured, and spotted on nitrocellulose (NC) membranes, followed by UV crosslinking and subsequent blocking with 5% non-fat milk. NC blots were then incubated with anti-5mC or 5hmC antibodies overnight at 4℃, followed by incubation with HRP-conjugated secondary antibodies and visualization with ECL Western Blotting Reagent (Pierce).

**Co-immunoprecipitation and immunoblotting assay**

For co-immunoprecipitation, cells expressing the proteins of interest were lysed in co-IP buffer (50 Mm Tris-Cl PH 7.5, 150 mM NaCl, 5.0 mM EDTA, 1.0% NP-40) supplemented with protease inhibitor cocktail (Roche). The cleared supernatant lysates were then incubated with specific antibodies and protein G agarose beads, or incubated with Anti-Flag M2 beads. The immunoprecipitates were denatured at 100 °C for 10 minutes in 2 × SDS-PAGE loading buffer, and then subjected to SDS-PAGE before blotting to PVDF membrane (Bio-Rad). PVDF blots were then blocked with 5% non-fat milk, incubated with specific primary antibodies and HRP-conjugated secondary antibodies, and finally visualized with ECL Western Blotting Reagent.

**Chromatin immunoprecipitation (ChIP) and Chip-seq data analysis**

ChIP experiments were performed as previously described([4](#_ENREF_4)) with modifications. For tissues, mice were anesthetized, brain were dissected and digested in 0.5% trypsin at 37°C, single cells filtrated and crosslinked in 1.0% formaldehyde. For cell lines, HEK293T/GT1-7 cells were crosslinked in 1.0% formaldehyde (Sigma) for 10 min at room temperature, followed by quenching in 125 mM glycine, then washed twice with ice-cold PBS, and resuspended with 270 µL lysis buffer (50 mM Tris-Cl PH 8.0, 10 mM EDTA, 1% SDS and protease inhibitor), after incubation on ice for 5 min, cells were sonicated with Bioruptor (UCD-300 ) for 15 cycles of 30s on ,30s off at high setting. Samples were centrifuged at 13,000 g for 10 min at 4°C,100 µL supernatant was diluted 10 times with ChIP dilution buffer ( 20 mM Tris-Cl PH 8.0, 0.01% SDS, 1.1% Triton X-100, 1.1 mM EDTA,167 mM NaCl) and incubated with 5 µg of control rabbit IgG or anti-MBD3 antibody at 4°C overnight. Samples were further incubated with 40 µL of Protein G beads at 4°C for 2 hr. The beads were washed thrice with Low Salt wash Buffer (20 mM Tris-Cl PH 8.0 ,150 mM NaCl, 0.1% SDS, 1% Triton X-100, 2 mM EDTA), three times with High Salt wash Buffer (20 mM Tris-Cl PH 8.0, 500 mM NaCl, 1% NP-40, 0.1% SDS, 2 mM EDTA), thrice with LiCl wash Buffer (20 mM Tris-Cl PH 8.0,500 mM LiCl, 1% NP-40, 1 mM EDTA, 1% deoxycholate),and thrice with TE buffer (100 mM Tris-Cl PH 8.0, 1 mM EDTA).Washed beads were resuspended with 500µL fresh elution buffer (1% SDS and 0.1 M sodium bicarbonate) and incubated at 65°C for 30 min. Eluted DNA was adjusted to 300 mM NaCl and incubated at 65°C for 4 hours, followed by incubation at 55°C for 1 hr with 50 ug proteinase K. DNA was purified using phenol-chloroform approach and subjected to ChIP-seq or ChIP-qPCR. Sequencing libraries were prepared with Truseq DNA kit or Nextera XT kit (following the manufacturer’s protocols) and sequenced using Illumina technologies at the National Center for Gene Research, Chinese Academy of Sciences.

As for ChIP-Seq analysis, sequencing reads were trimmed and then mapped against hg19 using Bowtie([5](#_ENREF_5)). Genomic regions bound by proteins were identified using MACS([6](#_ENREF_6)). Peaks annotation has been performed using the annotate Peaks.pl function of the Homer software package ([7](#_ENREF_7)). Bed tools were used to locate the overlapped and specific genomic regions for different proteins, with the bound motif identified by the RSAT peak-motifs tool([8](#_ENREF_8)). Our ChIP-Seq data was submited to the GEO repository (GSE102945).

Besides our data, 5 published MBD3 ChIP-Seq datasets and those 3 published on TET2 were analyzed to identify the common genes set they bound. The GEO numbers of these datasets were listed in **Table S5**

**Yeast Two-Hybrid Screen**

Yeast two hybrid screening was performed as described before, using human MKRN3 as the bait ([2](#_ENREF_2)). Positive colonies were subjected to survival test in SD-4 (deficient in *Ura*, *His*, *Leu* and *Trp*) medium, and also stained for β-glycosidase activity using X-Gal (Sigma). Identities of the hits were then determined by sequencing.

**GST pull-down Assays**

Purified Gst-MKRN3 (10 μg) ,MBD3-His and Glutathione Sepharose 4B were incubated at 4℃ overnight in 500ml of pull-down buffer (20 mM Tris-Cl, 100 mM NaCl, 5 mM MgCl2, 1 mM EDTA, 1 mM DTT, 0.5% (v/v) NP-40 and 10 μg/ml BSA, PH 7.5). The beads were then pelleted and washed for 3 times with the pull-down buffer (10min incubation at 4℃ for each washing). Then the recovered beads were boiled with protein loading buffer and finally analyzed by immunoblotting. GST Pull-down assays with MKRN3 and MBD3 fragments were performed following the same procedure.

***In Vitro* and *In Vivo* ubiquitination Assays**

*In vitro* ubiquitylation assays were performed as described previously([2](#_ENREF_2)). Briefly ATP, UB, E1(Uba1), E2(UBCH5A or UBCH7), E3(MKRN3), and MBD3 ,with USP2cc or not, were added in tubes,37℃ for 1 hour, then boiled and subjected to immunoblotting or mass analysis.

For *in vivo* ubiquitylation assays, cells were transfected with the indicated combination of the vectors. 48 hrs later, cells were harvested, lysed in buffer A ( 50 mM Tris-Cl PH 7.6, 150 mM NaCl,1% NP-40 ,1% sodium deoxychlate,1% SDS), then boiled 95℃ for 10min, diluted 10 times with buffer B ( 50 mM Tris-Cl PH 7.6,150 mM NaCl) and sonicated, followed by immunoprecipitation with specific antibodies at 4℃overnight, washed thrice with RIPA buffer (50 mM Tris-Cl PH 7.6,150 mM NaCl,1% NP-40 ,0.5% sodium deoxychlate, 0.1%SDS ), immunoprecipitates were then boiled in 1X SDS PAGE sampling buffer and subjected to immunoblotting analyses using anti-Ub or other relevant antibodies. As for tissue, firstly were homogenized by Precellys 24 in buffer A, and then followed the method above.

**Mass Spectra to map protein ubiquitination sites**

*In vitro* ubiquitylation assays of MBD3 or MKRN3 were performed firstly, and then subjected to mass spectra for ubiquitination sites analysis as described previously([2](#_ENREF_2)).

**Electrophoretic mobility shift assay (EMSA)**

The electrophoresis mobility shift assay (EMSA) was performed as previously described ([9](#_ENREF_9), [10](#_ENREF_10)) with some modifications. Probes corresponding to -1716 to -1541 bp of the human *GnRH1* gene were made by PCR using Platinum^®^Tag polymerase and NTPs containing either unmodified, methylated, or hydroxymethylated Dctp. The PCR primers sequences for probes construction were: Forward primer: 5’-TTCTTCAGCTTTGGGACTCAGAC-3’; Reverse primer: 5’-TGTATTACTCAGCATTCTCTAGA GG-3’. The probes were labeled with γ-^32^P at the 3’-end with T4 Polynucleotide Kinase (NEB) and purified by phenol-chloroform method. Recombinant human MBD3 or ubiquitylated MBD3 ( purified from In vitro ubiquitylation using anti-Flag beads, and eluted with Flag peptide ) was incubated with labeled probes in 5X EMSA Binding Buffer (Beyotime) at room temperature for 1 hour, and then the assay was performed on 6% polyacrylamide 0.5X TBE gels to resolve the DNA-protein complexes from the free probes. Gels were then dried and exposed to a phosphor-imager (Kodak) before visualization on a FLA 9000 Fuji scanner.

**Methylated DNA Immunoprecipitation (mDIP)**

MDIP was performed as previously described ([11](#_ENREF_11)). Briefly, genomic DNAs from the described tissues were extracted using the standard phenol – chloroform method firstly, and then sonicated to generate random fragments of sizes between 200 to 800bp. Subsequently, 10μg fragmented DNAs was denatured for 10 min at 95 °C and immunoprecipitated it overnight at 4 °C with anti-5hmC or anti-5mC antibodies in IP buffer (10 mM sodium phosphate (PH 7.0), 150 mM NaCl, 0.05% Triton X-100), incubated the mixture with protein G agarose beads for 2 h at 4 °C and washed five times with IP buffer. The immunoprecipitates on beads were further treated with proteinase K for 4 h at 55°C, subjected to extraction using phenol-chloroform approach. The resulted DNAs were then subjected to quantitative PCR analyses. Three independent experiments were performed.

**Blood Collection and Generation Immortalized Human B-Lymphocytes**

Under informed consents, the whole blood samples were collected from Central Precocious Puberty (CPP) proband with the MKRN3 mutation and her family members. After cell isolation in Lymphocyte separation medium (Qcbic S&T), human B-Lymphocytes were immortalized using Epstein-Barr virus (EBV) transformation approach. Briefly, the separated lymphocytes were cultured in fresh medium 1:1 EBV supernatant from B95-8 cells, supplied with 200ng/ml cyclosporine for 5-7 days; Medium was then replaced with fresh growth medium by letting the cells settle in the flask and carefully removing the upper medium. This cell feeding step was repeated several times in the next 2-3 weeks until transformation is achieved. Finally, the transformed lymphocyte cell line (LCL), also called lymphoblasts, and were obtained for further experiments.

**Statistics**

Data were analyzed by two tailed unpaired t-test, one-way ANOVA with Bonferroni post-hoc test, or Dennett’s post-hoc test using GraphPad Prism 7. *P < 0.05 was considered to be significant,** P < 0.01 was considered to be very significant.

**
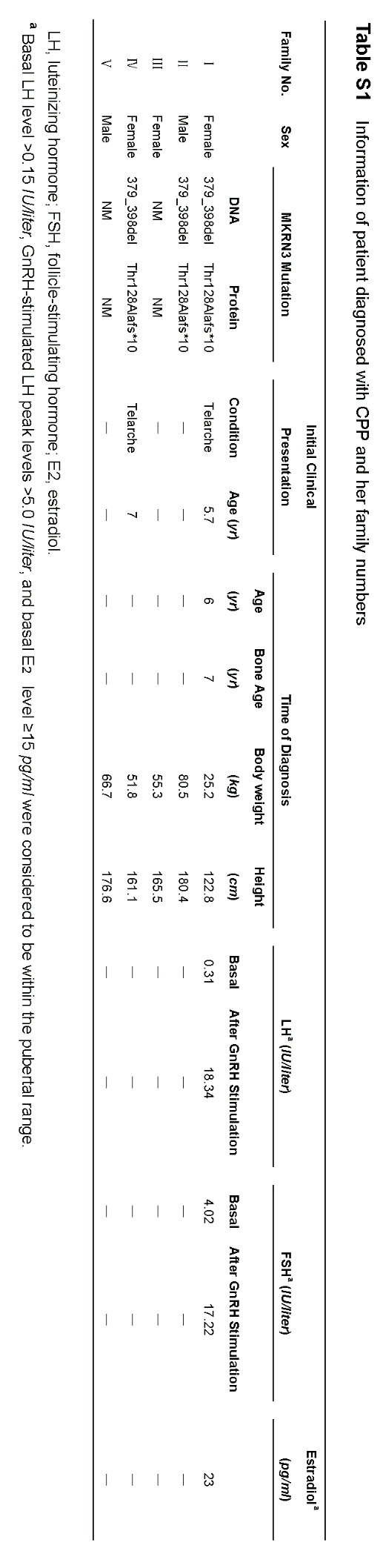
**

**Table S2** All plasmids used in this study

| Pdest32-MKRN3 | Prk5-His-UB |
| --- | --- |
| Pcdna3.0-MKRN3-Flag | Prk5-HA-UB(KO) |
| Pcdna3.0-MKRN3-3Xflag | Prk5-HA-UB(K6) |
| Pcdna3.0-MKRN3-3Xmyc | Prk5-HA-UB(K11) |
| Pcdna3.0-Mmkrn3-Flag | Prk5-HA-UB(K27) |
| Pdonr223-MBD3 | Prk5-HA-UB(K29) |
| Pcdna3.0-MBD3-HA | Prk5-HA-UB(K33) |
| Pcdna3.0-Flag-MKRN3_Pro161Argfs*10_ | Prk5-HA-UB(K48) |
| Pcdna3.0-Flag-MKRN3_Pro161Argfs*16_ | Prk5-HA-UB(K63) |
| Pcdna3.0-Flag-MKRN3_Ala162Glyfs*14_ | Prk5-HA-UB(K27R) |
| Pcdna3.0-Flag-MKRN3_Arg213Glyfs*73_ | Pgex4T-1- MKRN3 |
| Pcdna3.0-Flag-MKRN3_Gln226Thrfs*6_ | Pgex4T-1- MKRN3(1-125) |
| Pcdna3.0-Flag-MKRN3_Glu256Glyfs*36_ | Pgex4T-1- MKRN3(126-295) |
| Pcdna3.0-Flag-MKRN3_Cys340Gly_ | Pgex4T-1- MKRN3(296-380) |
| Pcdna3.0-Flag-MKRN3_Arg365Ser_ | Pgex4T-1- MKRN3(381-507) |
| Pcdna3.0-Flag-MKRN3_Tyr391*_ | Pet28a-Usp2cc |
| Pcdna3.0-Flag-MKRN3_Phe417Ile_ | Prfp-C-RS- shmMKRN3-1 |
| Pcdna3.0-Flag-MKRN3_His420Gln_ | Prfp-C-RS- shmMKRN3-2 |
| PX330-sgMBD3-1 | Prfp-C-RS- shmMKRN3-3 |
| PX330-sgMBD3-2 | PX330-sgMKRN3-1 |
| PX330-sgMBD3-3 | PX330-sgMKRN3-2 |
| PX330-sgMBD3-4 | PX330-sgMKRN3-3 |
| PX330-sgMBD3-5 | PX330-sgMKRN3-4 |
| PX330-sgMBD3-6 | PX330-sgMKRN3-5 |
| Pet28a-MBD3-6*His | PX330-sgMKRN3-6 |
| Pet28a-MBD3(1-90)-6*His | Pcdna3.0-MBD3_K142R_-HA |
| Pet28a-MBD3(91-291)-6*His | Pcdna3.0-MBD3_K157R_-HA |
| Pet28a-UBCH5A-6*His | Pcdna3.0-MBD3_K163R_-HA |
| Pet28a-UBCH5B-6*His | Pcdna3.0-MBD3_K216R_-HA |
| Pet28a-UBCH5C-6*His | Pcdna3.0-MBD3_K227R_-HA |
| Pet28a-UBCH7-6*His | Pcdna3.0-MBD3_K129,157,163,216,227R_-HA |
| Pet28a-UBCH13-6*His | PGL3-GnRH1-P356 |
| Pet28a-UBA1-6*His | PGL3-GnRH1-P1134 |
| Pegfp-C1-MKRN3 | PGL3-GnRH1-P3007 |
| Pcdna3.1(+)-MBD3-RFP-Flag | Pcdna3.0-MBD3-Flag |
| Pacyc-UB(HA)-UBA1(Flag)-UBCH7(V5) | Pcdna3.0-MBD(1-90)-Flag |
| Pacyc-UB(HA)-UBA1(Flag)-UBCH7(V5)-MKRN3 | Pcdna3.0-MBD3(91-291)-Flag |
| Pacyc-UB(HA)-UBA1(Flag)-UBCH5A(V5) | Pcdna3.0-Mtet2-HA |
| Pacyc-UB(HA)-UBA1(Flag)-UBCH5A(V5)-MKRN3 | Pcdna3.0-Mtet2(1-1098)-HA |
| Pet22b-MBD3-6*His | Pcdna3.0-Mtet2(1099-1912)-HA |
| Pgex4T-1-MKRN3_Cys340Gly_ | Pgex4T-1-MKRN3_Phe417Ile_ |
| Pgex4T-1-MKRN3_Arg365Ser_ | Pgex4T-1-MKRN3_His420Gln_ |

**Table S3** All antibodies used in this study

| **Antibody** | **Source** | **Company** | **Catalog** |
| --- | --- | --- | --- |
| MKRN3 | R | SIGMA | HPA029494 |
| MBD3 | R | Abcam | ab157464 |
| Ubiquitin | M | Santa Cruz | sc-8017 |
| TET1 | R | Absci | AB38243 |
| TET2 | R | Proteintech | [21207-1-AP](https://www.ptglab.com/products/TET2-Antibody-21207-1-AP.htm) |
| TET3 | R | Abcam | ab139311 |
| 5hmC | R | Active motif | 39791 |
| 5Mc | R | Abcam | ab10805 |
| GnRH1 | R | Thermo Fisher | PA1-121 |
| KISS1 | R | Proteintech | 18375-1-AP |
| NKB | R | Novus Biologicals | NB300-201 |
| GNRHR | R | Proteintech | 19950-1-AP |
| GAPDH | M | Proteintech | [60004-1-Ig](https://www.ptglab.com/products/GAPDH-Antibody-60004-1-Ig.htm) |
| β-Tublin | M | Proteintech | 11224-1-AP |
| Flag Tag | R | Proteintech | 20543-1-AP |
| Flag Tag | M | SIGMA | F3040 |
| HA Tag | R | SIGMA | SAB4300603 |
| His Tag | R | SIGMA | SAB1306085 |
| Myc Tag | R | SIGMA | SAB4301136 |
| Anti-Flag Affinity Gel | M | SIGMA | A4596 |
| Anti-HA Affinity Gel | M | SIGMA | E6779 |
| M，mouse R，Rabbit | | | |

**Table S4 Primers** used for quantitative PCR (qPCR), ChIP-qPCR or mDIP-qPCR in this study

| **qPCR** | |
| --- | --- |
| mGAPDH-F | AGAGTGTTTCCTCGTCCCGTAGACA |
| mGAPDH-R | CGTTGAATTTGCCGTGAGTGGAGTC |
| mGnRH1-F | GAAAGAGAAACACTGAACACTTGGT |
| mGnRH1-R | TCCTCTTCAATCAGACTTTCCAGAG |
| mMKRN3-F | AAGCGCATACTGGCATCAAG |
| mMKRN3-R | AGCCAACGGTCATCAGAGAA |
| mKiss1-F | CATCACTGGCTTTGGCGTCTTC |
| mKiss1-R | CTTGAGCTTGTGCCGTTGGAA |
| mTAC3-F | CCTCAGCTTGGCTTGGACCTTC |
| mTAC3-R | CTCAGCACTTTCAGCAATCCTTCCA |
| mL19-F | CTGAAGGTCAAAGGGAATGTG |
| mL19-R | GGACAGAGTCTTGATGATCTC |
| **ChIP-qPCR and EMSA** | |
| GNRH1-p-F1 | TTCAGTATAAAGCACCTTATCCAAGGAT |
| GNRH1-p-R1 | CAGCGATGATCTCATAATATCACTGAAATG |
| GNRH1-p-F2 (qPCR and EMSA) | TTCTTCAGCTTTGGGACTCAGAC |
| GNRH1-p-R2 (qPCR and EMSA) | TGTATTACTCAGCATTCTCTAGAGG |
| GNRH1-p-F3 | TGTAAATAACACGTCCACGGTTGC |
| GNRH1-p-R3 | GCAGTGGGAACAGAAAGATGAGAAA |
| mGnrh1-p-f1 | CCATTATTAAGACCTATTTTCTCCAGTTC |
| mGnrh1-p-r1 | CCACTTTCCTTTGTAGTTTGACGCT |
| mGnrh1-p-f2 | GTCACTGCAAAGTCCCTCTTCC |
| mGnrh1-p-r2 | CTGAGATCGGAAACATTGCTGGCA |
| mGnrh1-p-f3 | ATAGCACATGCACGAAGGTCAG |
| mGnrh1-p-r3 | GCTCAGTGGTTAGAGGTTGCTCC |
| **mDIP-qPCR** | |
| mGnRH1-mp-F | CCCTCTTCTGGTGTGTCTGAAGAC |
| mGnRH1-mp-R | CTGGAACCCACTTTGTAGACCAG |
| GNRH1-mp-F | GCCTAACCTCCCAGCCTACATC |
| GNRH1-mp-R | TGTATTACTCAGCATTCTCTAGAGG |

**Table S5** The GEO numbers of published MBD3 and TET2 ChIP-seq datasets re-analyzed in this study

| Title | GEO | Species |
| --- | --- | --- |
| MBD3 ChIP-seq | | |
| [MBD3_REP1](http://www.ncbi.nlm.nih.gov/geo/query/acc.cgi?acc=GSM1974448) | [GSM1974448](http://www.ncbi.nlm.nih.gov/geo/query/acc.cgi?acc=GSM1974448) | Homo sapiens |
| [MBD3_REP2](http://www.ncbi.nlm.nih.gov/geo/query/acc.cgi?acc=GSM1974449) | [GSM1974449](http://www.ncbi.nlm.nih.gov/geo/query/acc.cgi?acc=GSM1974449) | Homo sapiens |
| [MDA231_MBD3](http://www.ncbi.nlm.nih.gov/geo/query/acc.cgi?acc=GSM1089819) | [GSM1089819](http://www.ncbi.nlm.nih.gov/geo/query/acc.cgi?acc=GSM1089819) | Homo sapiens |
| [MCF-7_MBD3](http://www.ncbi.nlm.nih.gov/geo/query/acc.cgi?acc=GSM1089817) | [GSM1089817](http://www.ncbi.nlm.nih.gov/geo/query/acc.cgi?acc=GSM1089817) | Homo sapiens |
| [V5-MBD3-HELA_CHIP-SEQ](http://www.ncbi.nlm.nih.gov/geo/query/acc.cgi?acc=GSM1006708) | [GSM1006708](http://www.ncbi.nlm.nih.gov/geo/query/acc.cgi?acc=GSM1006708) | Homo sapiens |
| TET2 ChIP-seq | | |
| HT-TET2 CHIP-SEQ | GSM897576 | Homo sapiens |
| LNCAP CHIPED BY TET2 | GSM1613322 | Homo sapiens |
| HCT116 | GSM1152879 | Homo sapiens |

**SUPPLEMENTAL REFERENCES**

1. Cora, MC, Kooistra, L, Travlos, G. Vaginal Cytology of the Laboratory Rat and Mouse: Review and Criteria for the Staging of the Estrous Cycle Using Stained Vaginal Smears. *Toxicologic pathology*. 2015; **43**(6): 776-93.

2. Liu, Z, Chen, P, Gao, H*, et al.* Ubiquitylation of autophagy receptor Optineurin by HACE1 activates selective autophagy for tumor suppression. *Cancer Cell*. 2014; **26**(1): 106-20.

3. Komor, AC, Badran, AH, Liu, DR. CRISPR-Based Technologies for the Manipulation of Eukaryotic Genomes. *Cell*. 2017; **169**(3): 559.

4. Shimbo, T, Du, Y, Grimm, SA*, et al.* MBD3 localizes at promoters, gene bodies and enhancers of active genes. *PLoS genetics*. 2013; **9**(12): e1004028.

5. Langmead, B, Trapnell, C, Pop, M*, et al.* Ultrafast and memory-efficient alignment of short DNA sequences to the human genome. *Genome biology*. 2009; **10**(3): R25.

6. Zhang, Y, Liu, T, Meyer, CA*, et al.* Model-based analysis of ChIP-Seq (MACS). *Genome biology*. 2008; **9**(9): R137.

7. Heinz, S, Benner, C, Spann, N*, et al.* Simple combinations of lineage-determining transcription factors prime cis-regulatory elements required for macrophage and B cell identities. *Molecular cell*. 2010; **38**(4): 576-89.

8. Thomas-Chollier, M, Herrmann, C, Defrance, M*, et al.* RSAT peak-motifs: motif analysis in full-size ChIP-seq datasets. *Nucleic acids research*. 2012; **40**(4): e31.

9. Yildirim, O, Li, R, Hung, JH*, et al.* Mbd3/NURD complex regulates expression of 5-hydroxymethylcytosine marked genes in embryonic stem cells. *Cell*. 2011; **147**(7): 1498-510.

10. Shen, J, Sheng, X, Chang, Z*, et al.* Iron metabolism regulates p53 signaling through direct heme-p53 interaction and modulation of p53 localization, stability, and function. *Cell reports*. 2014; **7**(1): 180-93.

11. Weber, M, Davies, JJ, Wittig, D*, et al.* Chromosome-wide and promoter-specific analyses identify sites of differential DNA methylation in normal and transformed human cells. *Nature genetics*. 2005; **37**(8): 853-62.
