# Supplementary material for: MKRN3 regulates the epigenetic switch of mammalian puberty via ubiquitination of MBD3
Source: Natl Sci Rev. 2020 Feb 14;7(3):671–85. doi: 10.1093/nsr/nwaa023 (PMC8288866; doi:10.1093/nsr/nwaa023)
Supplement: nwaa023_Supplemental_Files [file nwaa023_supplemental_files.zip › Supplenmentary.pdf]

Fig S1

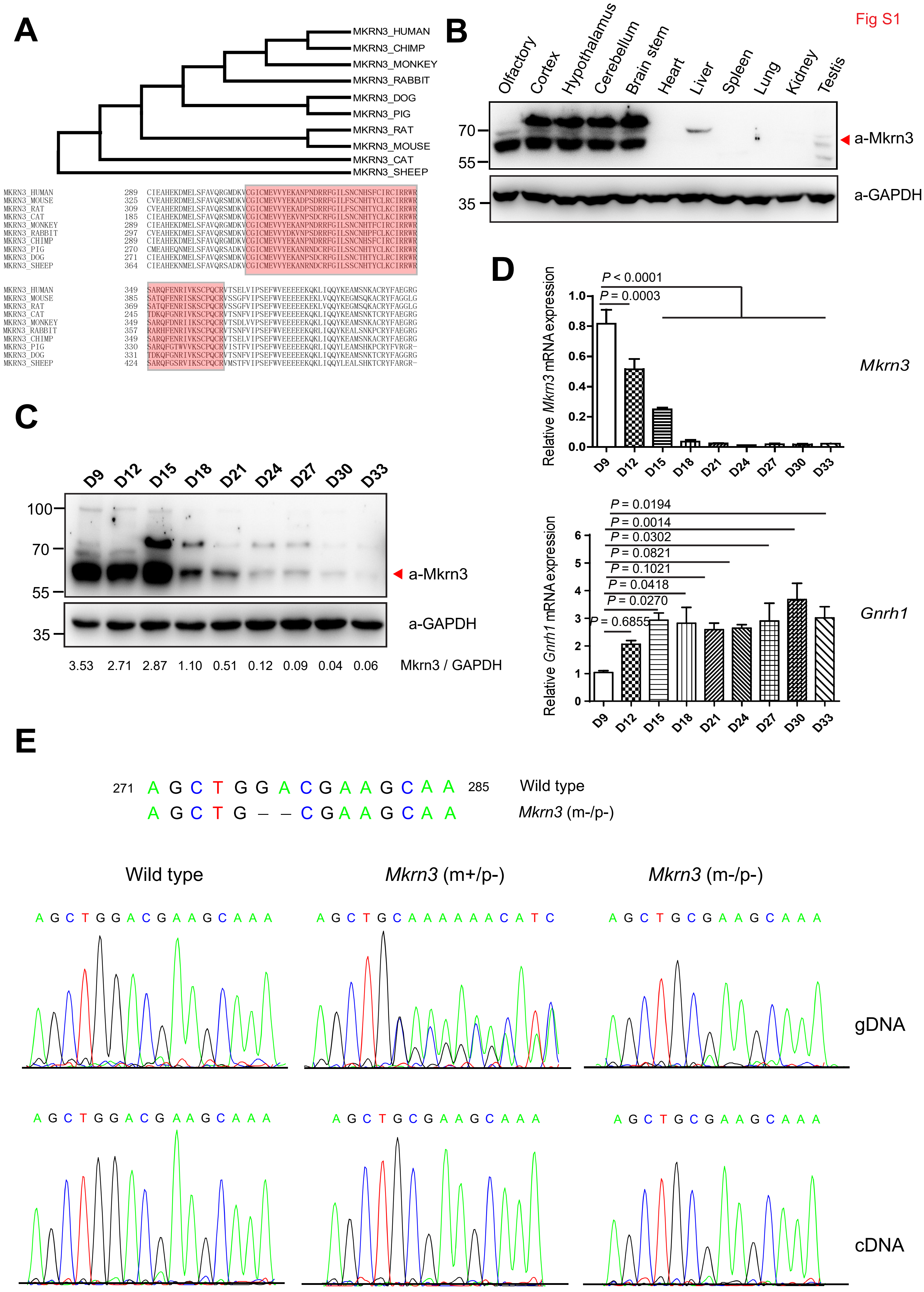

A
MKRN3_HUMAN
MKRN3_CHIMP
MKRN3_MONKEY
MKRN3_RABBIT
MKRN3_DOG
MKRN3_PIG
MKRN3_RAT
MKRN3_MOUSE
MKRN3_CAT
MKRN3_SHEEP
B
Olfactory
Cortex
Hypothalamus
Cerebellum
Brain stem
Heart
Liver
Spleen
Lung
Kidney
Testis
70
55
35
a-Mkrn3
a-GAPDH
C
D9 D12 D15 D18 D21 D24 D27 D30 D33
100
70
55
35
a-Mkrn3
a-GAPDH
3.53 2.71 2.87 1.10 0.51 0.12 0.09 0.04 0.06
Mkrn3 / GAPDH
D
P < 0.0001
P = 0.0003
Relative Mkrn3 mRNA expression
Mkrn3
P = 0.0194
P = 0.0014
P = 0.0302
P = 0.0821
P = 0.1021
P = 0.0418
P = 0.0270
P = 0.6855
Relative Gnrh1 mRNA expression
Gnrh1
E
271 A G C T G G A C G A A G C A A 285 Wild type
A G C T G – – C G A A G C A A Mkrn3 (m-/p-)
Wild type
Mkrn3 (m+/p-)
Mkrn3 (m-/p-)
A G C T G G A C G A A G C A A A
A G C T G C A A A A A A C A T C
A G C T G C G A A G C A A A
gDNA
A G C T G G A C G A A G C A A A
A G C T G C G A A G C A A A
A G C T G C G A A G C A A A
cDNA

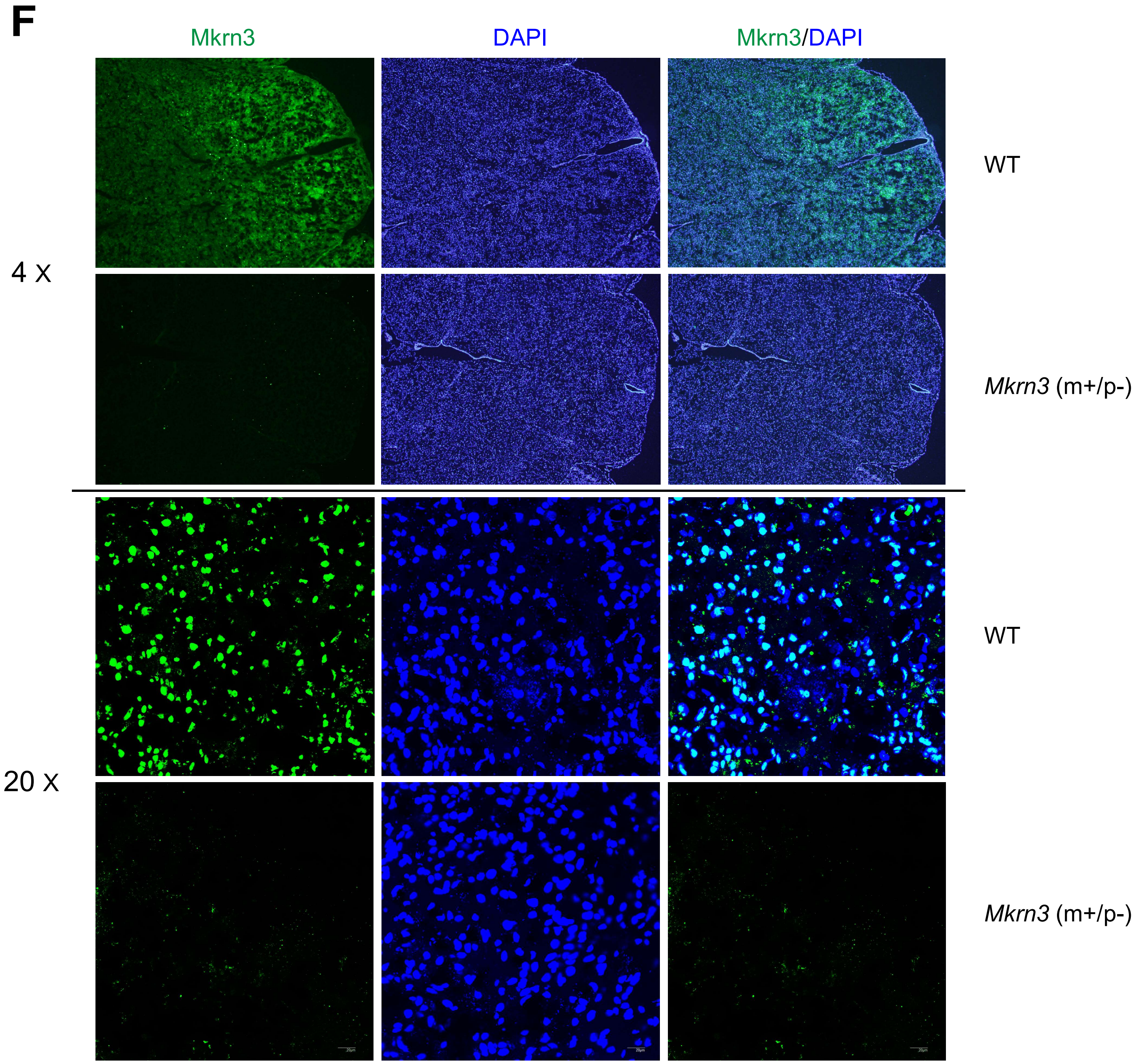
F
Mkrn3
DAPI
Mkrn3/DAPI
4 X
20 X
WT
Mkrn3 (m+/p-)
WT
Mkrn3 (m+/p-)

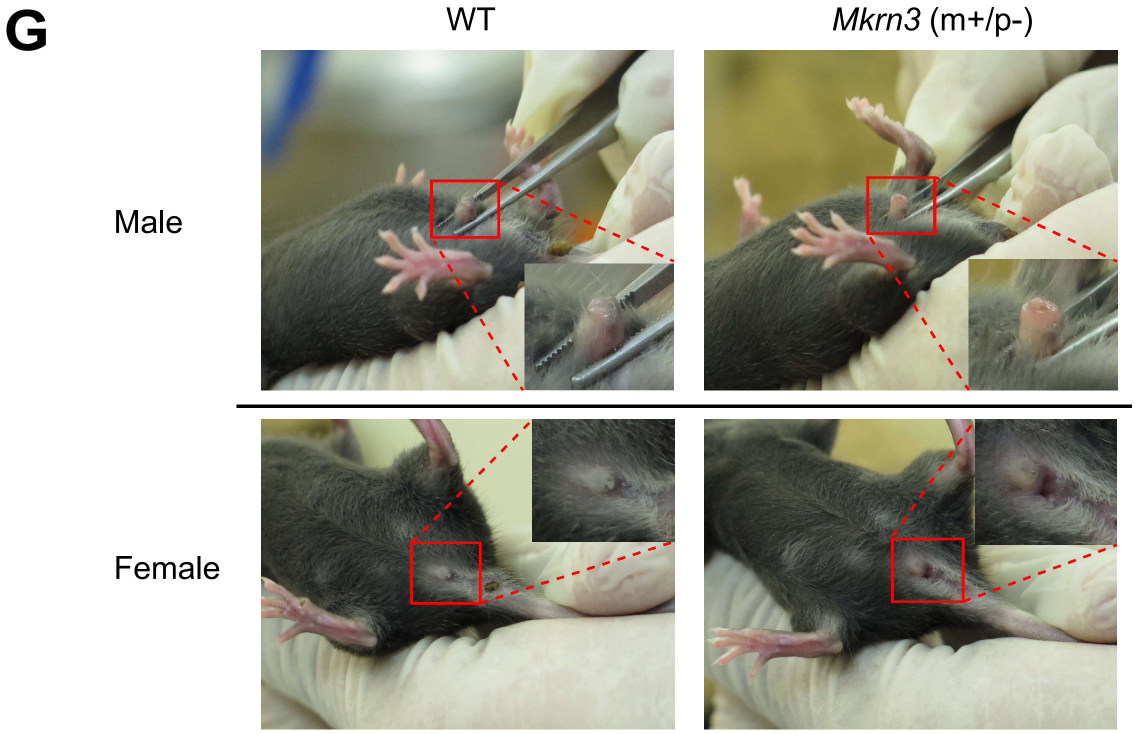
G
WT
Mkrn3 (m+/p-)
Male
Female

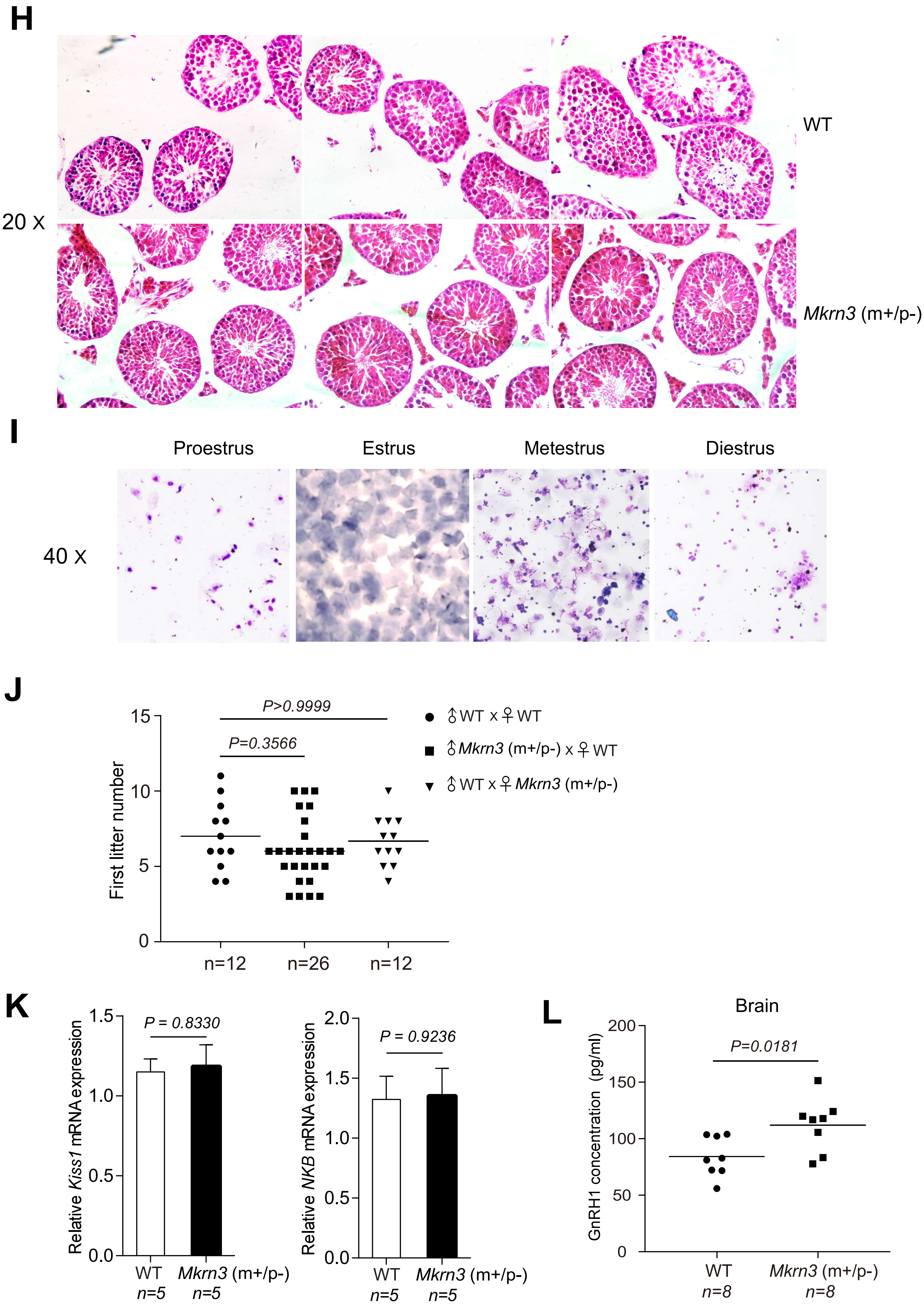
H
20 X
WT
Mkrn3 (m+/p-)
I
Proestrus
Estrus
Metestrus
Diestrus
40 X
J
P>0.9999
P=0.3566
First litter number
15
10
5
0
n=12
n=26
n=12
♂WT x ♀WT
♂Mkrn3 (m+/p-) x ♀WT
♂WT x ♀Mkrn3 (m+/p-)
K
P = 0.8330
Relative Kiss1 mRNA expression
1.5
1.0
0.5
0.0
WT
n=5
Mkrn3 (m+/p-)
n=5
P = 0.9236
Relative NKB mRNA expression
2.0
1.5
1.0
0.5
0.0
WT
n=5
Mkrn3 (m+/p-)
n=5
L
Brain
P=0.0181
GnRH1 concentration (pg/ml)
200
150
100
50
0
WT
n=8
Mkrn3 (m+/p-)
n=8

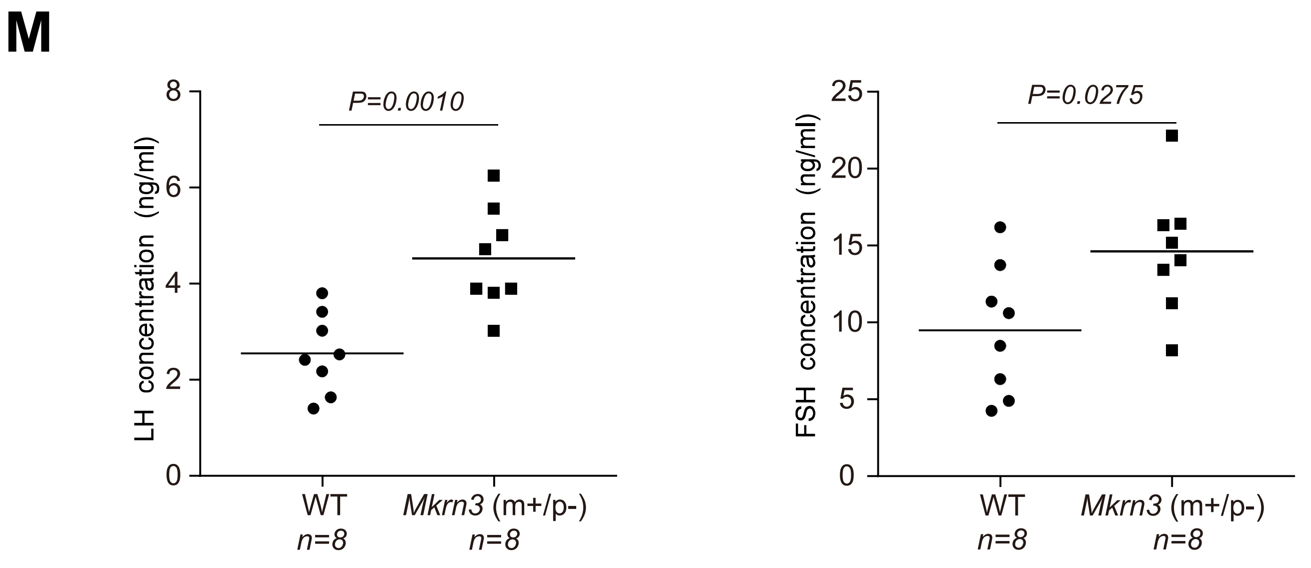
M
P=0.0010
LH concentration (ng/ml)
8
6
4
2
0
WT
n=8
Mkrn3 (m+/p-)
n=8
P=0.0275
FSH concentration (ng/ml)
25
20
15
10
5
0
WT
n=8
Mkrn3 (m+/p-)
n=8

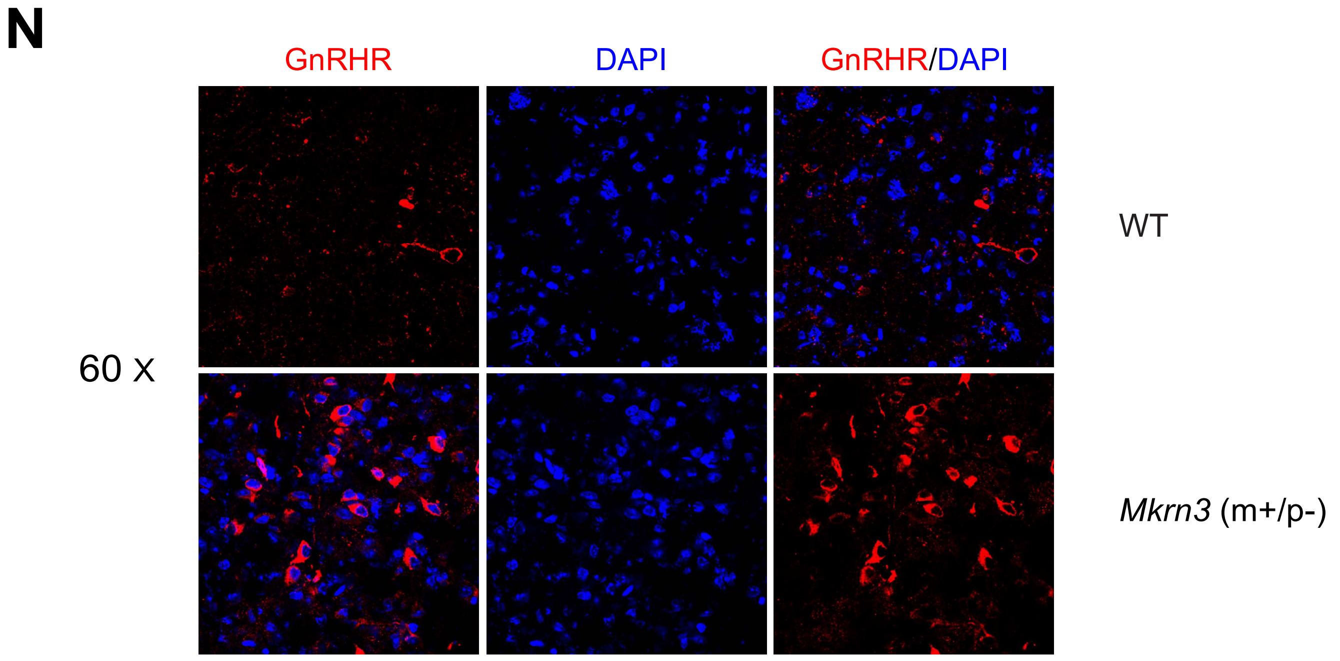
N
GnRHR
DAPI
GnRHR/DAPI
60 X
WT
Mkrn3 (m+/p-)

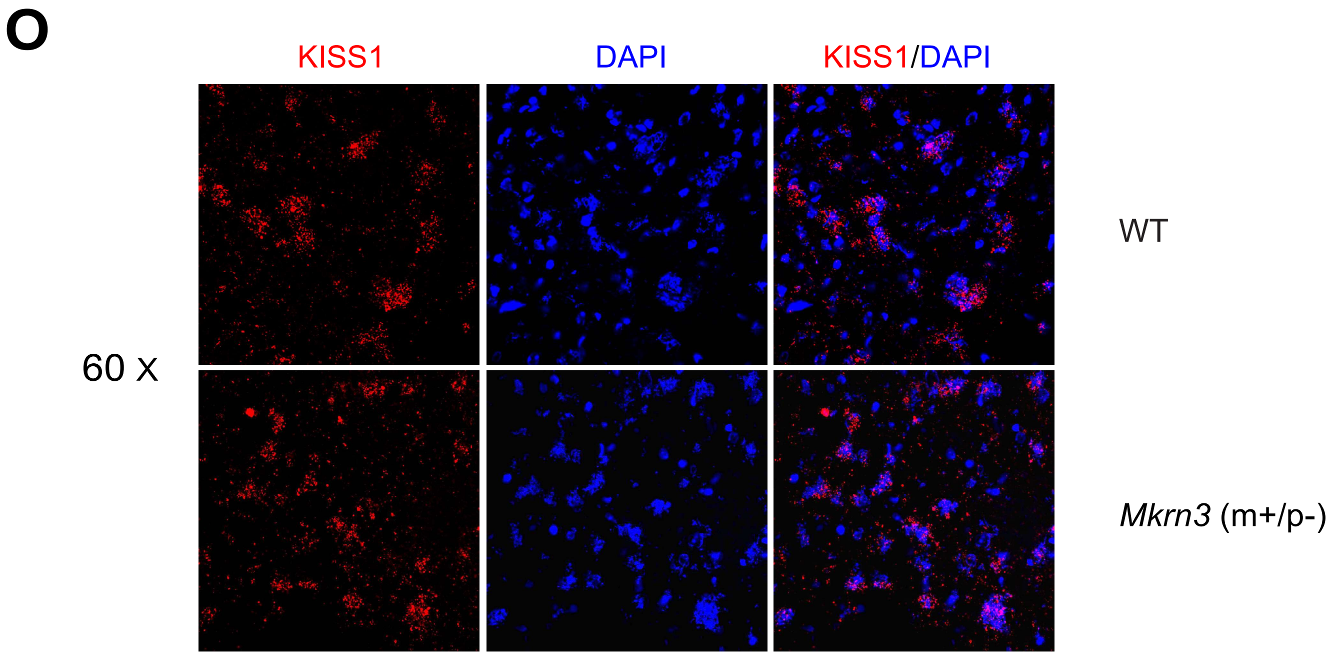
O
KISS1
DAPI
KISS1/DAPI
60 X
WT
Mkrn3 (m+/p-)

P

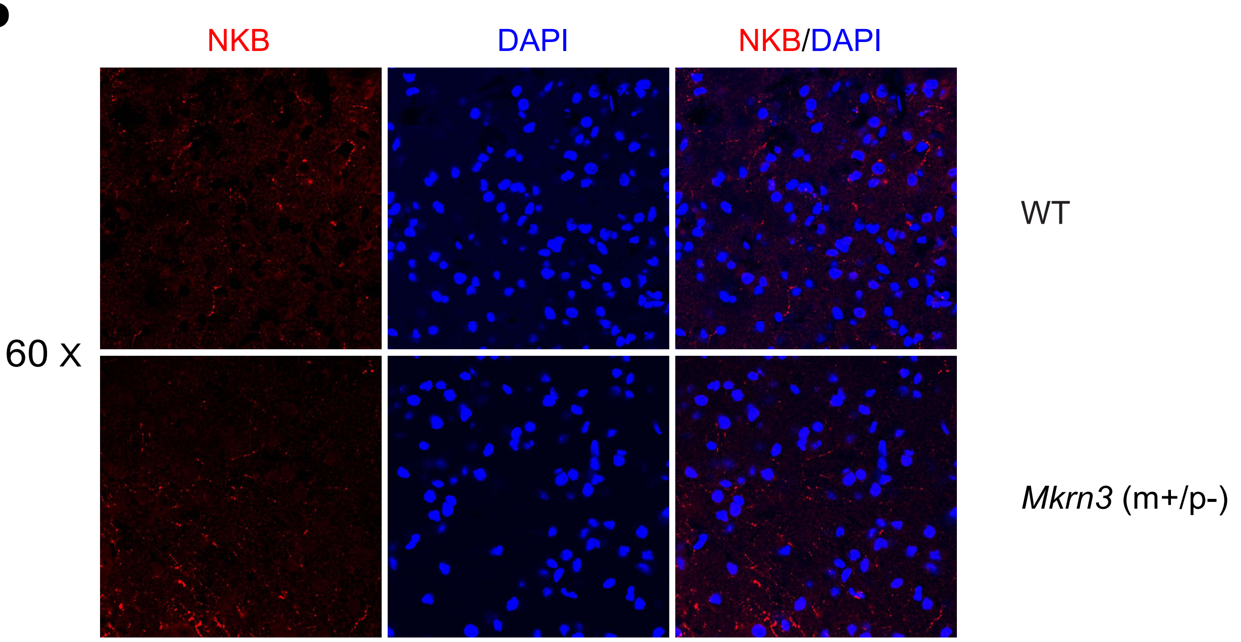
NKB
DAPI
NKB/DAPI
60 X
WT
Mkrn3 (m+/p-)

Q

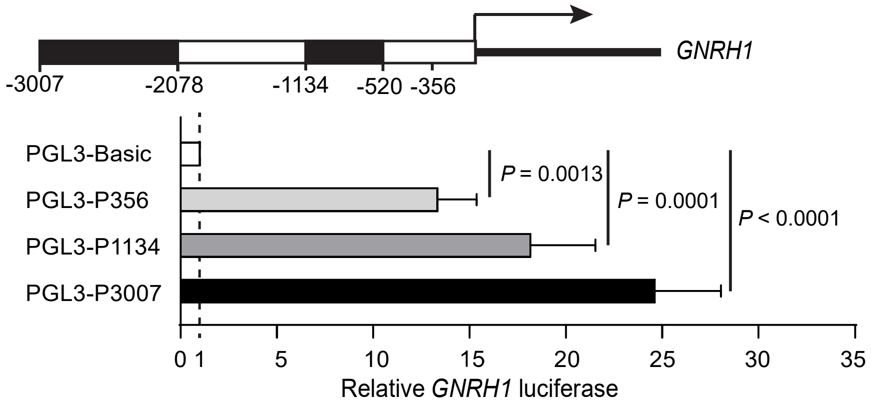
GNRH1
-3007
-2078
-1134
-520
-356
PGL3-Basic
PGL3-P356
PGL3-P1134
PGL3-P3007
P = 0.0013
P = 0.0001
P < 0.0001
0 1
5
10
15
20
25
30
35
Relative GNRH1 luciferase

R

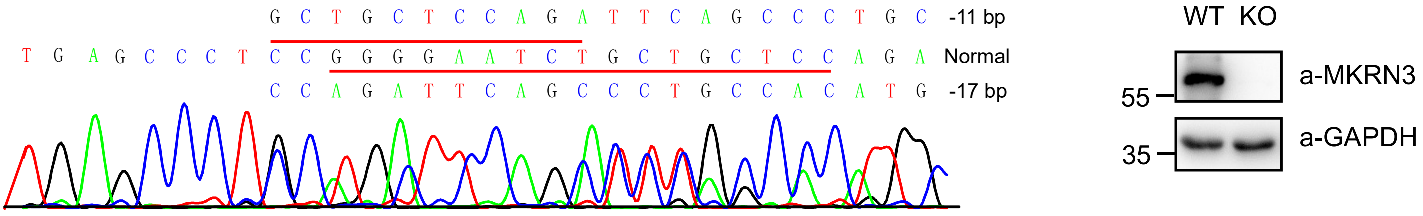
G C T G C T C C A G A T T C A G C C C T G C
-11 bp
T G A G C C C T C C G G G G A A T C T G C T G C T C C A G A
Normal
C C A G A T T C A G C C C T G C C A C A T G
-17 bp
WT
KO
55
35
a-MKRN3
a-GAPDH

S

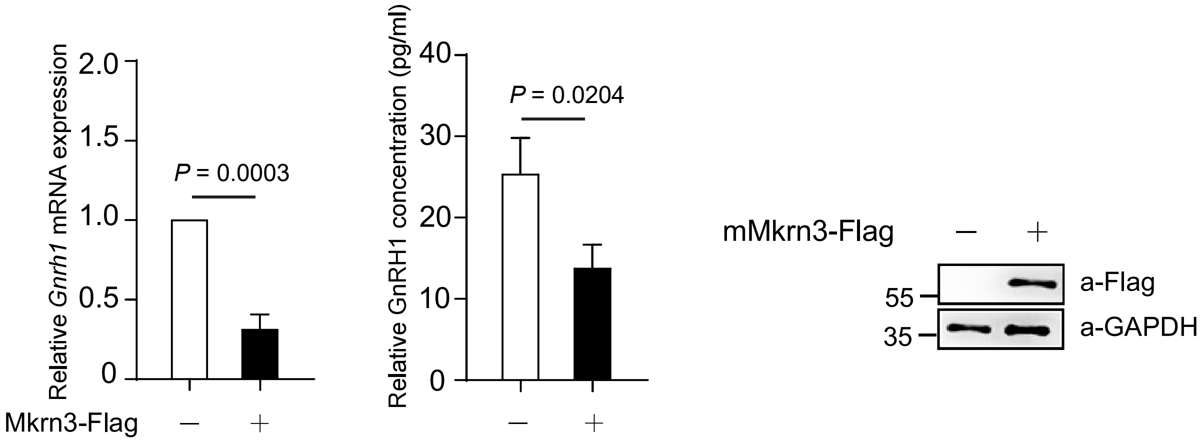
Relative Gnrh1 mRNA expression
2.0
1.5
1.0
0.5
0
P = 0.0003
Mkrn3-Flag
—
+
Relative GnRH1 concentration (pg/ml)
40
30
20
10
0
P = 0.0204
—
+
mMkrn3-Flag
—
+
55
35
a-Flag
a-GAPDH

Fig S2

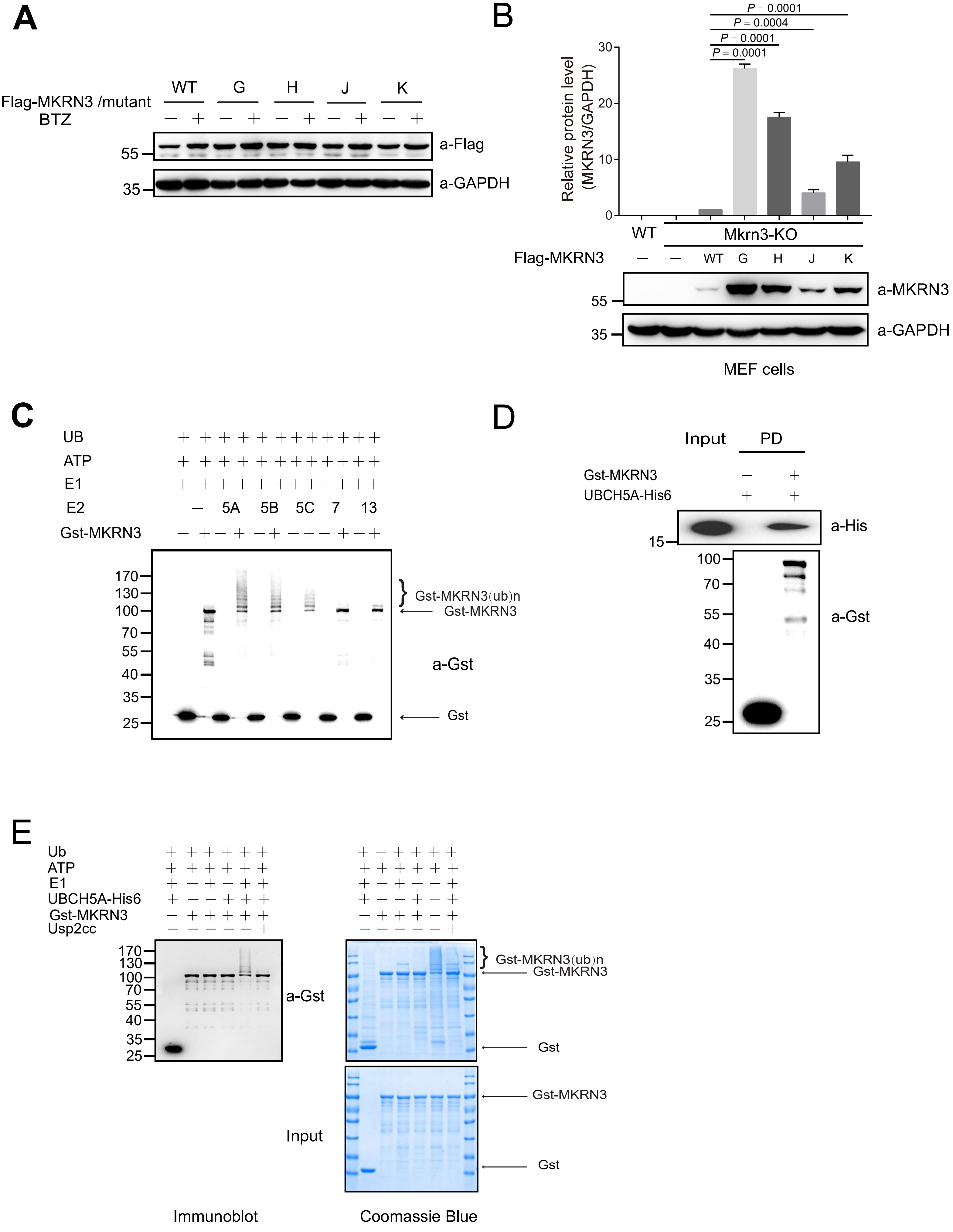
A
Flag-MKRN3 /mutant
BTZ
WT G H J K
— + — + — + — + — +
55
35
a-Flag
a-GAPDH
B
P = 0.0001
P = 0.0004
P = 0.0001
P = 0.0001
Relative protein level (MKRN3/GAPDH)
30
20
10
0
WT
Mkrn3-KO
Flag-MKRN3
— — WT G H J K
a-MKRN3
a-GAPDH
55
35
MEF cells
C
UB
ATP
E1
E2
— 5A 5B 5C 7 13
Gst-MKRN3
170
130
100
70
55
40
35
25
Gst-MKRN3(ub)n
Gst-MKRN3
a-Gst
Gst
D
Input
PD
Gst-MKRN3
UBCH5A-His6
a-His
15
100
70
55
40
35
25
a-Gst
E
Ub
ATP
E1
UBCH5A-His6
Gst-MKRN3
Usp2cc
170
130
100
70
55
40
35
25
a-Gst
Gst-MKRN3(ub)n
Gst-MKRN3
Gst
Input
Gst-MKRN3
Gst
Immunoblot
Coomassie Blue

Fig S3

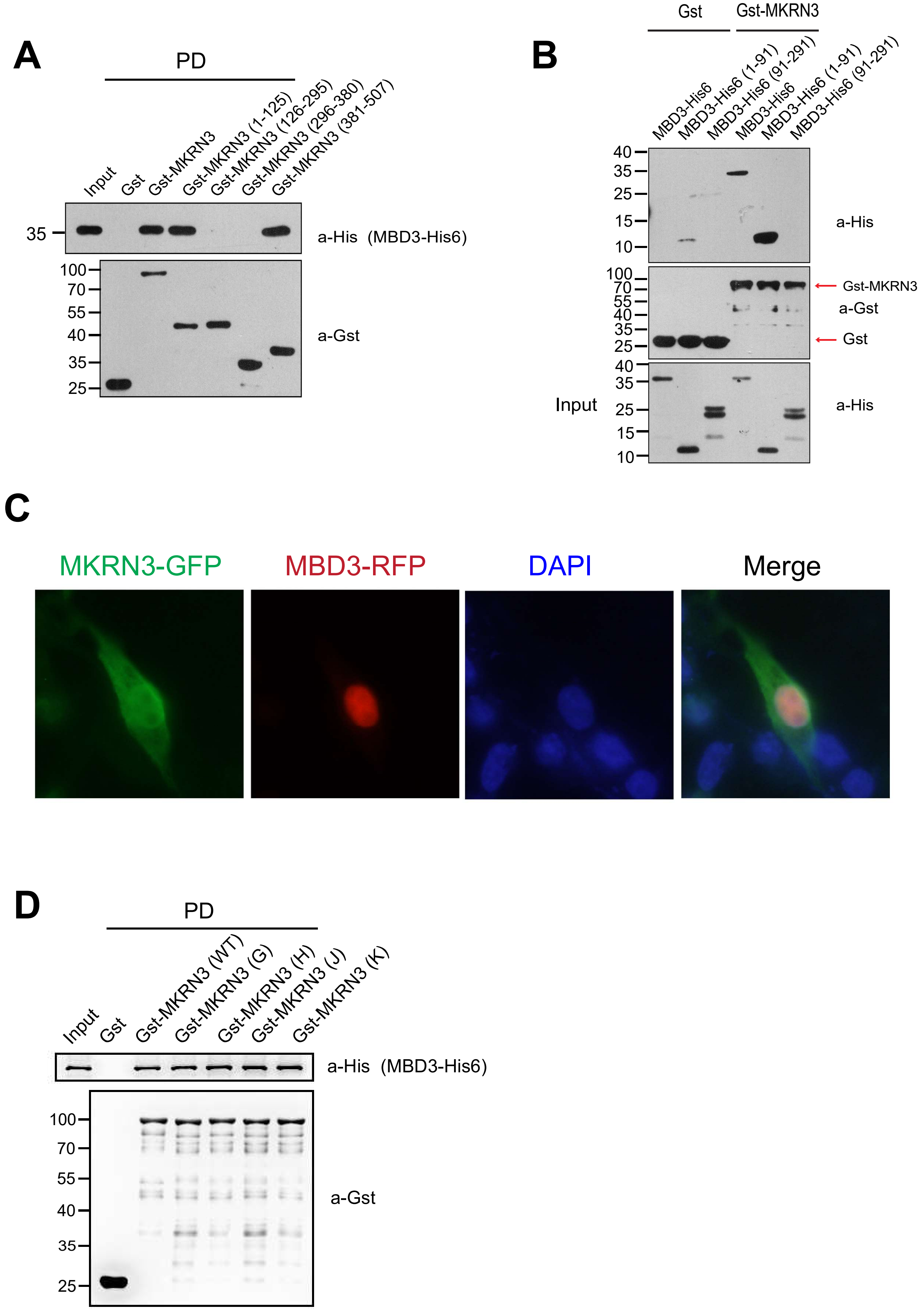
A
PD
Input
Gst
Gst-MKRN3
Gst-MKRN3 (1-125)
Gst-MKRN3 (126-295)
Gst-MKRN3 (296-380)
Gst-MKRN3 (381-507)
35
a-His (MBD3-His6)
100
70
55
40
35
25
a-Gst
B
Gst
Gst-MKRN3
MBD3-His6
MBD3-His6 (1-91)
MBD3-His6 (91-291)
MBD3-His6
MBD3-His6 (1-91)
MBD3-His6 (91-291)
40
35
25
15
10
a-His
100
70
55
40
35
25
Gst-MKRN3
a-Gst
Gst
Input
a-His
C
MKRN3-GFP
MBD3-RFP
DAPI
Merge
D
PD
Input
Gst
Gst-MKRN3 (WT)
Gst-MKRN3 (G)
Gst-MKRN3 (H)
Gst-MKRN3 (J)
Gst-MKRN3 (K)
a-His (MBD3-His6)
100
70
55
40
35
25
a-Gst

Fig S4

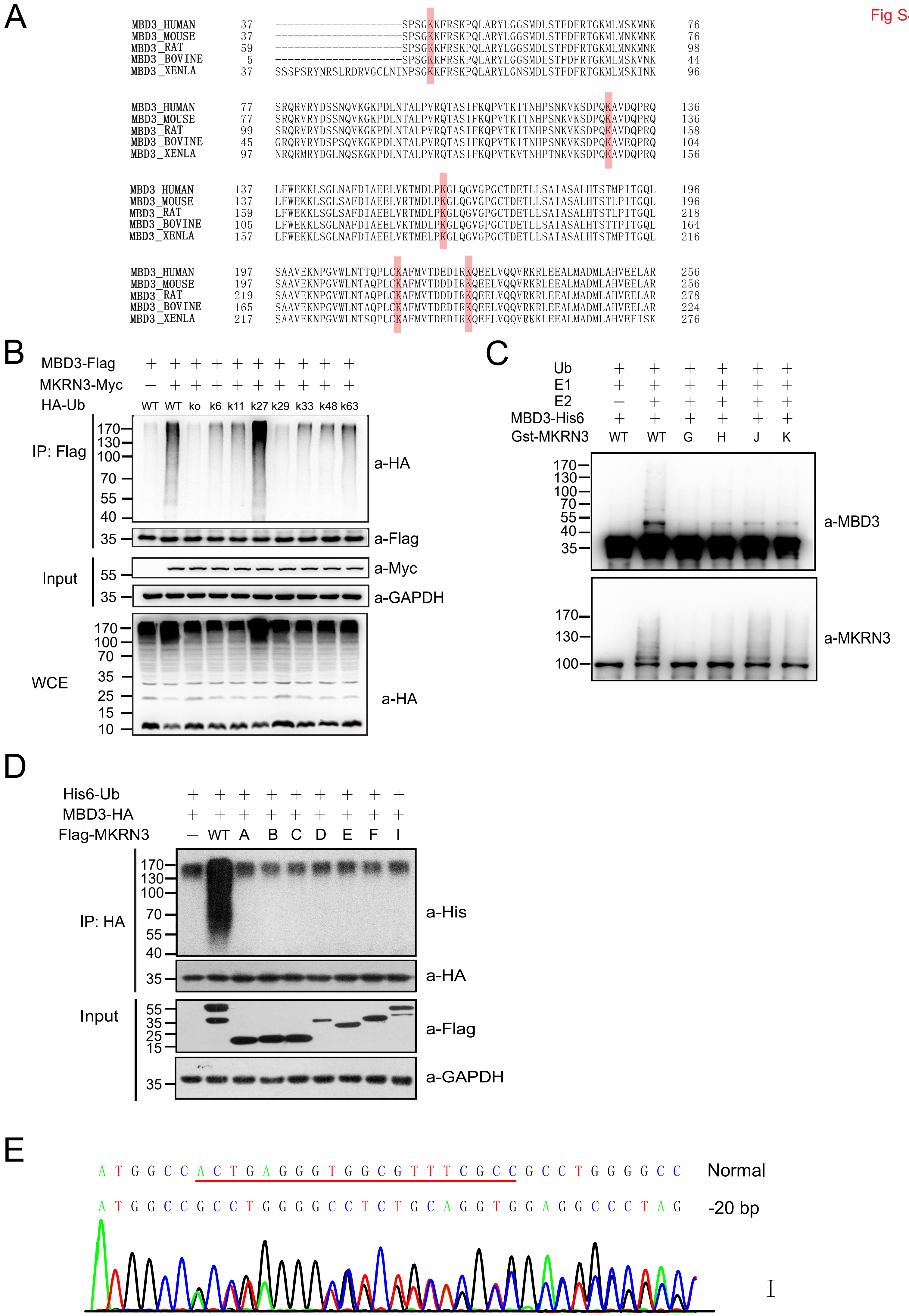
A
MBD3_HUMAN 37 --------------------SPSGKKFRSKPQLARYLGGSMDLSTFDFRTGKMLMSKMNK 76
MBD3_MOUSE 37 --------------------SPSGKKFRSKPQLARYLGGSMDLSTFDFRTGKMLMNKMNK 76
MBD3_RAT 59 --------------------SPSGKKFRSKPQLARYLGGSMDLSTFDFRTGKMLMNKMNK 98
MBD3_BOVINE 5 --------------------SPSGKKFRSKPQLARYLGGSMDLSTFDFRTGKMLMSKVNK 44
MBD3_XENLA 37 SSSPSRYNRSLRDRVGCLNINPSGKKFRSKPQLARYLGNSMDLSTFDFRTGKMLMSKINK 96
MBD3_HUMAN 77 SRQRVRYDSSNQVKGKPDLNTALPVRQTASIFKQPVTKITNHPSNKVKSDPQKAVDQPRQ 136
MBD3_MOUSE 77 SRQRVRYDSSNQVKGKPDLNTALPVRQTASIFKQPVTKITNHPSNKVKSDPQKAVDQPRQ 136
MBD3_RAT 99 SRQRVRYDSSNQVKGKPDLNTALPVRQTASIFKQPVTKITNHPSNKVKSDPQKAVDQPRQ 158
MBD3_BOVINE 45 GRQRVRYDSPSQVKGKPDLNTALPVRQTASIFKQPVTKITNHPSNKVKSDPQKAVEQPRQ 104
MBD3_XENLA 97 NRQRMRYDGLNQSKGKPDLNTALPVRQTASIFKQPVTKVTNHPTNKVKSDPQKAVDQPRQ 156
MBD3_HUMAN 137 LFWEKKLSGLNAFDIAEELVKTMDLPKGLQGVGPGCTDETLLSAIASALHTSTMPITGQL 196
MBD3_MOUSE 137 LFWEKKLSGLSAFDIAEELVRTMDLPKGLQGVGPGCTDETLLSAIASALHTSTLPITGQL 196
MBD3_RAT 159 LFWEKKLSGLSAFDIAEELVRTMDLPKGLQGVGPGCTDETLLSAIASALHTSTLPITGQL 218
MBD3_BOVINE 105 LFWEKKLSGLNAFDIAEELVKTMDLPKGLQGVGPGCTDETLLSAIASALHTSTTPITGQL 164
MBD3_XENLA 157 LFWEKKLSGLNAFDIAEELVKTMELPKGLQGVGPGCTDETLLSAIASALHTSTMPITGQL 216
MBD3_HUMAN 197 SAAVEKNPGVWLNTTQPLCKAFMVTDEDIRKQEELVQQVRKRLEEALMADMLAHVEELAR 256
MBD3_MOUSE 197 SAAVEKNPGVWLNTAQPLCKAFMVTDDDIRKQEELVQQVRKRLEEALMADMLAHVEELAR 256
MBD3_RAT 219 SAAVEKNPGVWLNTAQPLCKAFMVTDDDIRKQEELVQQVRKRLEEALMADMLAHVEELAR 278
MBD3_BOVINE 165 SAAVEKNPGVWLNTAQPLCKAFMVTDEDIRKQEELVQQVRKRLEEALMADMLAHVEELAR 224
MBD3_XENLA 217 SAAVEKNPGVWLNTSQPLCKAFMVTDEDIRKQEELVQQVRKKLEEALMADMLAHVEEISK 276
B
MBD3-Flag + + + + + + + + + +
MKRN3-Myc – + + + + + + + + +
HA-Ub WT WT ko k6 k11 k27 k29 k33 k48 k63
IP: Flag
170 130 100 70 55 40
a-HA
35
a-Flag
Input
55
a-Myc
35
a-GAPDH
WCE
170 100 70 35 25 15 10
a-HA
C
Ub + + + + + +
E1 + + + + + +
E2 – + + + + +
MBD3-His6 + + + + + +
Gst-MKRN3 WT WT G H J K
170 130 100 70 55 40 35
a-MBD3
170 130 100
a-MKRN3
D
His6-Ub + + + + + + + + +
MBD3-HA + + + + + + + + +
Flag-MKRN3 – WT A B C D E F I
IP: HA
170 130 100 70 55 40
a-His
35
a-HA
Input
55 35 25 15
a-Flag
35
a-GAPDH
E
A T G G C C A C T G A G G G T G G C G T T T C G C C G C C T G G G G C C
Normal
A T G G C C G C C T G G G G C C T C T G C A G G T G G A G G C C C T A G
-20 bp

Fig S5

A

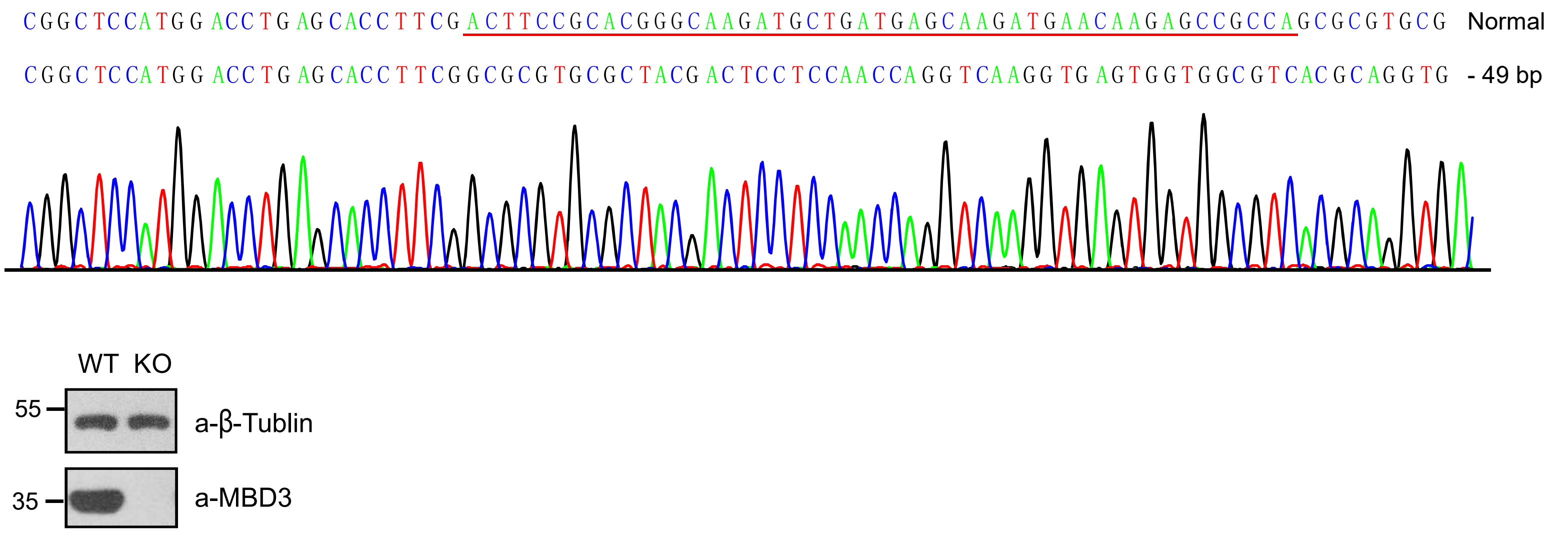

CGGCTCCATGG ACCTGAGCACCTTCGACTTCCGCACGGGCAAGATGCTGATGAGCAAGATGAACAAGAGCCGCCAGCGCGTGCG Normal
CGGCTCCATGG ACCTGAGCACCTTCGGCGCGTGCGCTACGACTCCTCCAACCAGGTCAAGGTGAGTGGTGGCGTCACGCAGGTG - 49 bp
WT KO
55
a-β-Tublin
35
a-MBD3

Fig S6

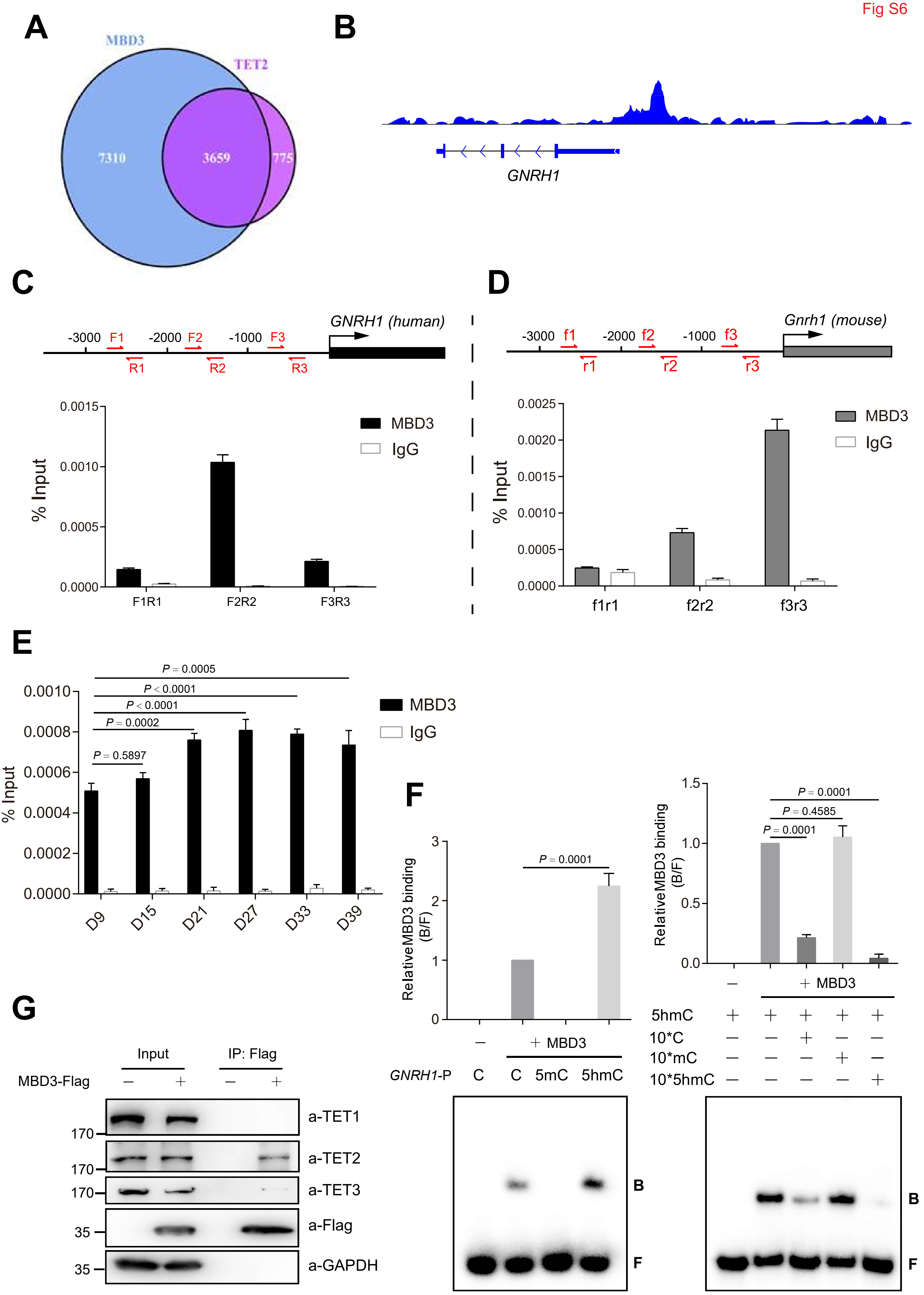
A
MBD3
TET2
7310
3659
775
B
GNRH1
C
GNRH1 (human)
-3000 F1 -2000 F2 -1000 F3
R1 R2 R3
% Input
MBD3
IgG
F1R1 F2R2 F3R3
D
Gnrh1 (mouse)
-3000 f1 -2000 f2 -1000 f3
r1 r2 r3
% Input
MBD3
IgG
f1r1 f2r2 f3r3
E
P = 0.0005
P < 0.0001
P < 0.0001
P = 0.0002
P = 0.5897
% Input
MBD3
IgG
D9 D15 D21 D27 D33 D39
F
P = 0.0001
Relative MBD3 binding (B/F)
– + MBD3
GNRH1-P C C 5mC 5hmC
B
F
P = 0.0001
P = 0.4585
P = 0.0001
Relative MBD3 binding (B/F)
– + MBD3
5hmC
10*C
10*mC
10*5hmC
B
F
G
Input
IP: Flag
MBD3-Flag
a-TET1
a-TET2
a-TET3
a-Flag
a-GAPDH
170
170
170
35
35

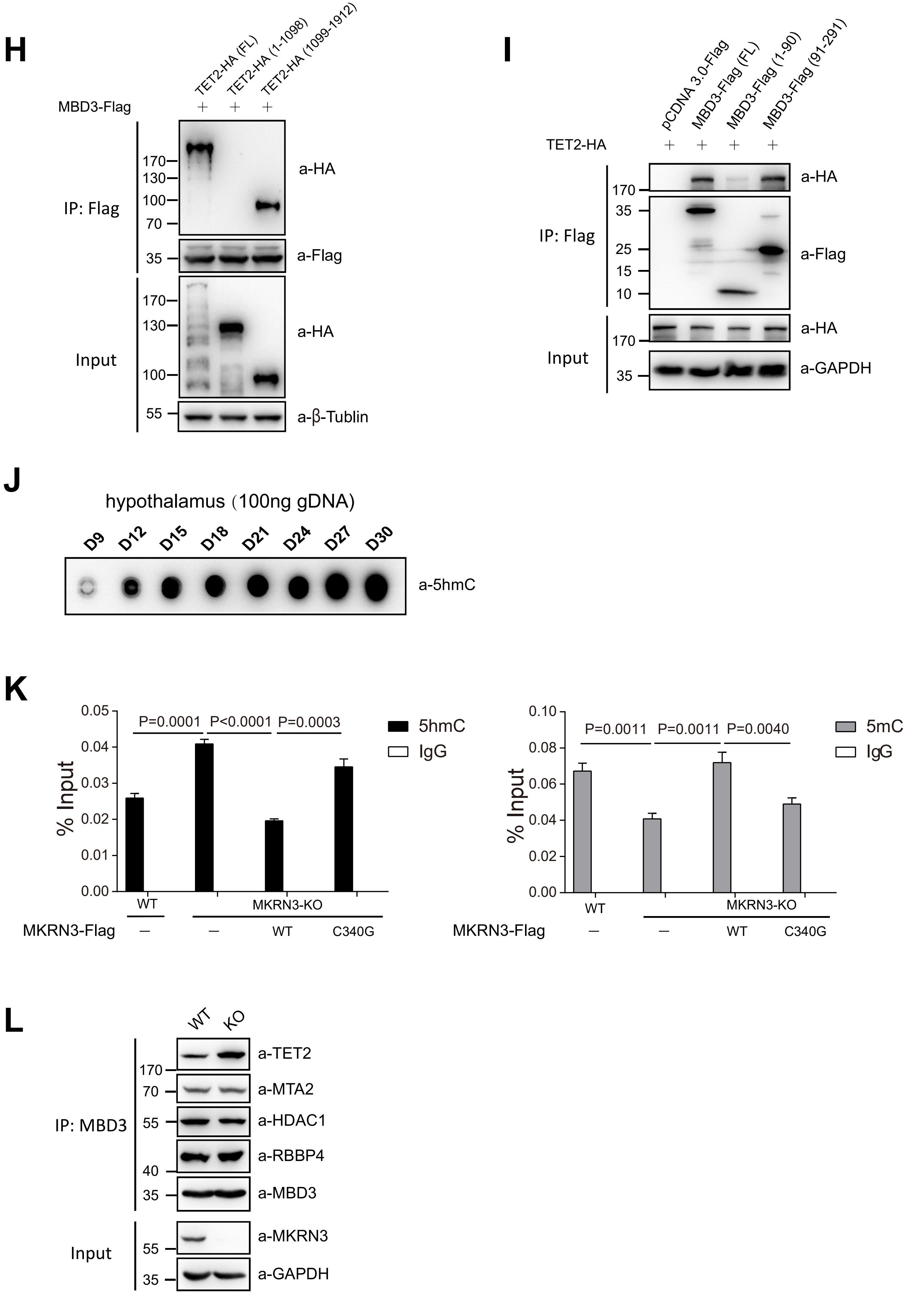
H
TET2-HA (FL)
TET2-HA (1-1098)
TET2-HA (1099-1912)
MBD3-Flag
IP: Flag
Input
a-HA
a-Flag
a-β-Tublin
170
130
100
70
35
55
I
pCDNA 3.0-Flag
MBD3-Flag (FL)
MBD3-Flag (1-90)
MBD3-Flag (91-291)
TET2-HA
IP: Flag
Input
a-HA
a-Flag
a-GAPDH
170
35
25
15
10
J
hypothalamus (100ng gDNA)
D9
D12
D15
D18
D21
D24
D27
D30
a-5hmC
K
% Input
P=0.0001
P<0.0001
P=0.0003
5hmC
IgG
WT
MKRN3-KO
MKRN3-Flag
WT
C340G
P=0.0011
P=0.0011
P=0.0040
5mC
L
WT
KO
IP: MBD3
Input
a-TET2
a-MTA2
a-HDAC1
a-RBBP4
a-MBD3
a-MKRN3
a-GAPDH
170
70
55
40
35
